# Lyssavirus M protein degrades neuronal microtubules by reprogramming mitochondrial metabolism

**DOI:** 10.1128/mbio.02880-23

**Published:** 2024-02-13

**Authors:** Yueming Yuan, An Fang, Haoran Wang, Caiqian Wang, Baokun Sui, Jianqing Zhao, Zhen F. Fu, Ming Zhou, Ling Zhao

**Affiliations:** 1 State Key Laboratory of Agricultural Microbiology, Huazhong Agricultural University, Wuhan, China; 2 Key Laboratory of Preventive Veterinary Medicine of Hubei Province, College of Veterinary Medicine, Huazhong Agricultural University, Wuhan, China; 3 Frontiers Science Center for Animal Breeding and Sustainable Production, Wuhan, China; 4 Hubei Hongshan Laboratory, Wuhan, China; Princeton University, Princeton, New Jersey, USA

**Keywords:** lyssavirus, neuron degeneration, mitochondria metabolism

## Abstract

Infection with neurotropic viruses may result in changes in host behavior, which are closely associated with degenerative changes in neurons. The lyssavirus genus comprises highly neurotropic viruses, including the rabies virus (RABV), which has been shown to induce degenerative changes in neurons, marked by the self-destruction of axons. The underlying mechanism by which the RABV degrades neuronal cytoskeletal proteins remains incomplete. In this study, we show that infection with RABV or overexpression of its M protein can disrupt mitochondrial metabolism by binding to Slc25a4. This leads to a reduction in NAD^+^ production and a subsequent influx of Ca^2+^ from the endoplasmic reticulum and mitochondria into the cytoplasm of neuronal cell lines, activating Ca^2+^-dependent proteinase calpains that degrade α-tubulin. We further screened the M proteins of different lyssaviruses and discovered that the M protein of the dog-derived RABV strain (DRV) does not degrade α-tubulin. Sequence analysis of the DRV M protein and that of the lab-attenuated RABV strain CVS revealed that the 57th amino acid is vital for M-induced microtubule degradation. We generated a recombinant RABV with a mutation at the 57th amino acid position in its M protein and showed that this mutation reduces α-tubulin degradation *in vitro* and axonal degeneration *in vivo*. This study elucidates the mechanism by which lyssavirus induces neuron degeneration.

**IMPORTANCE:**

Previous studies have suggested that RABV (rabies virus, the representative of lyssavirus) infection induces structural abnormalities in neurons. But there are few articles on the mechanism of lyssavirus’ effect on neurons, and the mechanism of how RABV infection induces neurological dysfunction remains incomplete. The M protein of lyssavirus can downregulate cellular ATP levels by interacting with Slc25a4, and this decrease in ATP leads to a decrease in the level of NAD^+^ in the cytosol, which results in the release of Ca^2+^ from the intracellular calcium pool, the endoplasmic reticulum, and mitochondria. The presence of large amounts of Ca^2+^ in the cytoplasm activates Ca^2+^-dependent proteases and degrades microtubule proteins. The amino acid 57 of M protein is the key site determining its disruption of mitochondrial metabolism and subsequent neuron degeneration.

## INTRODUCTION

Studies have shown that several viral infections are associated with degenerative changes in neurons (1). Lyssavirus is strictly neurophilic, and its retrograde axonal infiltration into the central nervous system rapidly infects neurons and causes neural axonal injury (2). To date, there is no effective drug against the pathogens that cause these deadly diseases. Previous studies have suggested that RABV (the representative of lyssavirus) infection induces structural abnormalities in neurons (3, 4), but there are few articles on the mechanism of lyssavirus’ effect on neurons, and the mechanism of how RABV infection induces neurological dysfunction remains incomplete. Lyssavirus is composed of five structural proteins: nucleoprotein (N), phosphoprotein (P), matrix protein (M), glycoprotein (G), and polymerase protein (L). The M protein is the smallest of the five structural proteins, consisting of 256 amino acids and encoding four domains: N-terminal domain (NTD), central domain (CD), C-terminal domain (CTD), and membrane-proximal domain (MPD). M protein is involved in the interaction between the capsid and the vesicle membrane and facilitates the movement of viral particles to the cell surface and egress through its PPxY structural domain (5, 6). It has recently been reported to interact with ATP6V1A to facilitate viral uncoating during viral entry into host cells (7). The M protein is responsible for regulating the transcription and replication balance of viral genes, increasing replication efficiency by inhibiting transcriptional activity (8, 9). It has also been reported to function as an immune escape agent by inhibiting the inflammatory signaling pathway of host cells (10, 11).

Solute carrier family 25 member 4 (Slc25a4), also called the adenine nucleotide translocase 1 (ANT1), is predominantly expressed in the heart, skeletal muscle, and brain (12). Slc25a4 is localized to the inner mitochondrial membrane and imports ADP into the mitochondrial matrix and exports ATP (13). Mitochondria from the Slc25a4-deficient mouse muscle secreted only about 5%–10% of the ATP secreted by wild-type muscle mitochondria, suggesting an important role for Slc25a4 in mitochondrial ATP production (14). Slc25a4 is also involved in the composition of the mitochondria membrane permeability and the mitochondrial uncoupling by acting as a proton transporter (15, 16). It has been reported that several viral proteins can interact with Slc25a4 to regulate mitochondria function. For example, latent membrane protein 1 (LMP1) encoded by Epstein–Barr virus (EBV) can interact with Slc25a4 and inhibit the opening of mitochondrial membrane permeability transition pore (mPTP) (17). Porcine β-defensin 2 (PBD-2) peptide encoded by swine influenza virus (SIV) binds Slc25a4 and thereby inhibits SIV-induced cell apoptosis (18).

Self-destruction of axons and dendrites in neurons is closely related to neuron degeneration (19). A recent report showed that the level of NAD^+^ in neurons determines the degeneration under RABV infection, and Sterile Alpha and TIR Motif-containing 1 (SARM1), a gene that plays a key role in axon degradation (20), is critical for neuron degeneration induced by RABV infection (4). SARM1 can continuously consume NAD^+^ (21, 22), and the decrease of the NAD^+^ level in neurons will lead to the increase of Ca^2+^ concentration, which will activate Ca^2+^-dependent proteinase calpains and cause the degradation of axon skeleton proteins, and ultimately lead to the self-destruction of axon dendrites (23–27). However, the mechanism of how RABV causes this phenomenon has not yet been elucidated.

In the present study, we found that the M protein of the RABV CVS strain of lyssavirus can downregulate cellular ATP levels by interacting with Slc25a4, and this decrease in ATP leads to a decrease in the level of NAD^+^ in the cytosol, which results in the release of Ca^2+^ from the intracellular calcium pool, the endoplasmic reticulum (ER), and mitochondria. The presence of large amounts of Ca^2+^ in the cytoplasm activates Ca^2+^-dependent proteases and degrades microtubule proteins. We also found that the M protein of the RABV DRV strain does not cause microtubule degradation. By screening of different mutants, we identified the 57th amino acid of CVS-M as the key site determining its disruption of mitochondrial metabolism. Finally, we infected transgenic mice expressing fluorescent proteins in neurons with recombinant viruses of CVS, DRV, and CVS-M-G57E and determined that the 57th amino acid of CVS-M determines whether the virus induces degenerative changes in neuronal axons.

## RESULTS

### Rabies virus matrix protein decreases intracellular ATP and NAD^+^ levels and interacts with Slc25a4

**Fig 1:**
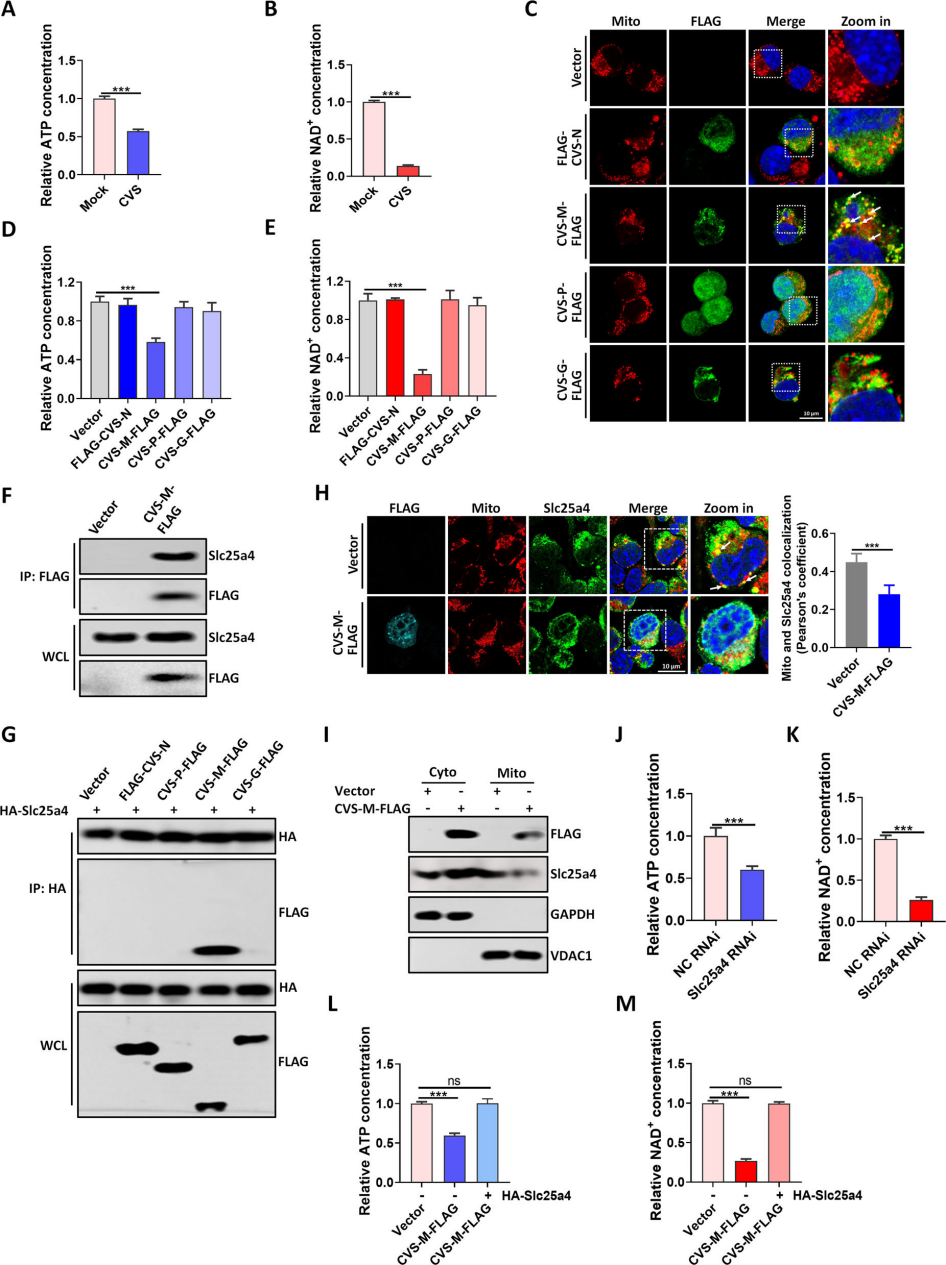
RABV matrix protein disrupts mitochondrial metabolism and interacts with Slc25a4. (**A**) N2a cells were mock infected or infected with CVS at multiplicity of infection (MOI)of 1 for 36 hours (h); the level of ATP was tested in the experimental groups, quantified as the ratio of (ATP concentration)/(ATP concentration of control group) × 100%. (**B**) N2a cells were mock infected or infected with CVS (MOI = 1) for 36 h; the level of NAD^+^ was tested in the experimental groups, quantified as the ratio of (NAD^+^ concentration)/(NAD^+^ concentration of control group) × 100%. (**C**) N2a cells were transfected with pCAGGS-FLAG (Vector), FLAG-CVS-N, CVS-M-FLAG, CVS-P-FLAG, or CVS-G-FLAG plasmids for 36 h. Cells were fixed and observed for colocalization of mitochondria with the indicated FLAG-tagged proteins. White boxes indicate the enlarged sections of images. White arrows indicate the colocalization between mitochondria with the CVS-M-FLAG. Scale bar, 10 µm. (**D, E**) N2a cells were transfected as in **C**; the levels of ATP (**D**) and NAD^+^ (**E**) were measured. (**F**) N2a cells were transfected with Vector or CVS-M-FLAG for 36 h; cell lysates were subjected to co-immunoprecipitation (Co-IP) and analyzed by western blotting (WB). (**G**) HA-Slc25a4 was co-expressed with pCAGGS-FLAG (Vector), FLAG-CVS-N, CVS-M-FLAG, CVS-P-FLAG, or CVS-G-FLAG plasmids for 36 h; cell lysates were subjected to Co-IP and analyzed by WB. (**H**) N2a cells were transfected with Vector or CVS-M-FLAG for 36 h. Cells were fixed and analyzed for colocalization of mitochondria with the endogenous Slc25a4. White boxes indicate the enlarged sections of images. White arrows indicate the colocalization between mitochondria with the endogenous Slc25a4. Scale bar, 10 µm. Pearson’s correlation coefficient analysis between mitochondria (Mito) and Slc25a4 is shown in the right panel. At least 30 cells were included for each group. (**I**) N2a cells were transfected with Vector or CVS-M-FLAG for 36 h; mitochondria were isolated and analyzed by WB. (**J, K**) N2a cells were transfected with siRNAs for Slc25a4 for 36 h; the levels of ATP (**J**) and NAD^+^ (**K**) were measured. (**L, M**) N2a cells were transfected with Vector or CVS-M-FLAG or cotransfected CVS-M-FLAG and HA-Slc25a4 for 36 h; the levels of ATP (**L**) and NAD^+^ (**M**) were measured. Error bars, mean ± SD of three experiments. Statistical analysis of comparisons between groups was carried out by Student’s *t*-test (*P < 0.05, **P < 0.01, and ***P < 0.001; ns, not significant). Western blot data are representative of three independent experiments. See also Fig. S1.

To investigate the impact of RABV infection on the cellular energy metabolism of neuron cells, we infected mouse neuroblastoma (N2a) cells with the RABV CVS strain and observed significant reductions in both ATP and NAD^+^ levels following infection (Fig. 1A and B). To identify which viral protein of the CVS strain is responsible for reducing cellular energy metabolism, we generated plasmids encoding FLAG-tagged structural proteins of RABV, including the nucleoprotein (N), phosphoprotein (P), matrix protein (M), and glycoprotein (G), using the pCAGGS-FLAG expression vector (named FLAG-CVS-N, CVS-P-FLAG, CVS-M-FLAG, and CVS-G-FLAG). Following transfection of these plasmids into N2a cells, we observed co-localization of CVS-M-FLAG with mitochondria, the cellular energy metabolism center (Fig. 1C). Furthermore, overexpression of CVS-M-FLAG protein significantly reduced intracellular ATP and NAD^+^ levels (Fig. 1D and E). Collectively, these results suggest that the M protein of RABV can disrupt mitochondrial energy metabolism.

To elucidate the mechanism by which CVS-M reduces intracellular ATP and NAD^+^ levels, we expressed CVS-M-FLAG as bait and performed immunoprecipitation (IP) followed by mass spectrometry (MS) analysis. Our results revealed an interaction between Slc25a4 and CVS-M-FLAG, which was further confirmed by Co-IP (Fig. 1F). We also confirmed that CVS-P, CVS-M, or CVS-G was not interacted with Slc25a4 (Fig. 1G).

Slc25a4 acts as a master regulator of mitochondrial energy output by functioning as an ADP-ATP antiporter (16). To further investigate how CVS-M affects cellular energy metabolism, we used WB and showed that CVS-M expression did not affect the expression level of Slc25a4 (Fig. S1A). The confocal microscopy analysis showed that CVS-M affected the localization of endogenous Slc25a4, and its co-localization with mitochondria was significantly reduced by CVS-M overexpression (Fig. 1H). We then performed mitochondrial isolation experiments, and the WB results showed that Slc25a4 on mitochondria was decreased upon CVS-M overexpression. These experiments suggest that CVS-M expression leads to the translocation of Slc25a4 (Fig. 1I).

In addition, we knocked down Slc25a4 using siRNA (Fig. S1B) and observed significant reductions in cellular ATP and NAD^+^ levels following knockdown (Fig. 1J and K). Next, we cloned mouse Slc25a4 with an HA tag into the pCAGGS expression vector (named HA-Slc25a4) and co-expressed CVS-M-FLAG and HA-Slc25a4 in N2a cells. We found that expression of HA-Slc25a4 increased cellular ATP and NAD^+^ levels that were reduced by CVS-M-FLAG (Fig. 1L and M), indicating that Slc25a4 expression attenuates the reduction of ATP and NAD^+^ levels induced by CVS-M. Collectively, these results suggest that CVS-M may inhibit ATP and NAD^+^ production by interacting with the ADP/ATP translocase Slc25a4.

### CVS-M expression or CVS infection leaks Ca^2+^ from intracellular Ca^2+^ pools and causes deformation of ER

**Fig 2:**
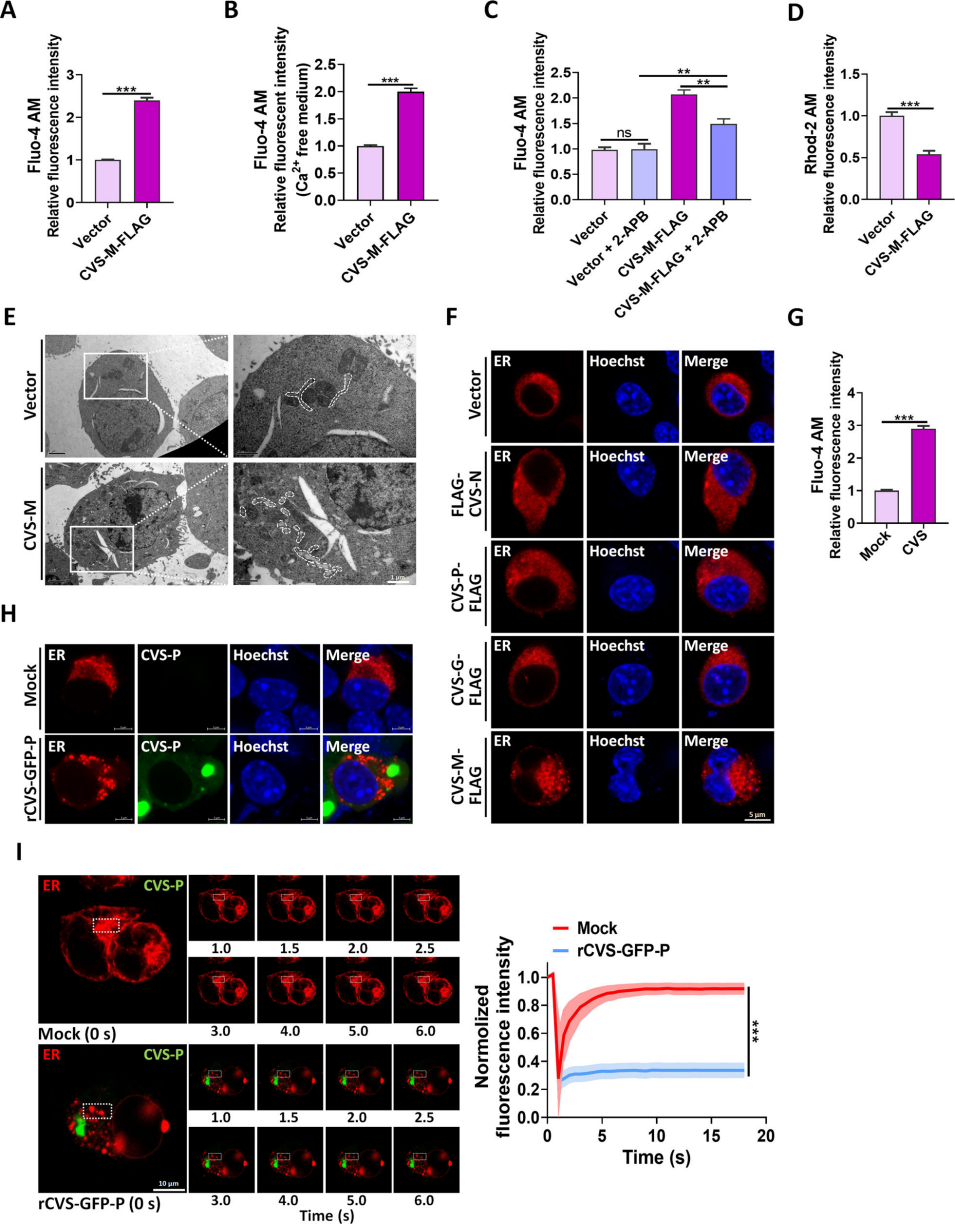
Expression of CVS-M or infection with CVS leads to leakage of Ca^2+^ from intracellular Ca^2+^ pools and morphology change of the ER. (**A**) N2a cells were transfected with Vector or CVS-M-FLAG for 36 h and then stained with Fluo-4 AM. The fluorescence signals of Fluo-4 AM were acquired under 488 nm excitation light, quantified as the ratio of (Ca^2+^ concentration)/(Ca^2+^ concentration of control group) × 100%. (**B**) N2a cells were cultured in Ca^2+^-free medium and transfected with Vector or CVS-M-FLAG for 36 h and then stained with Fluo-4 AM, and Ca^2+^ concentrations were measured. (**C**) N2a cells were cultured in Ca^2+^-free medium and transfected with Vector or CVS-M-FLAG and then were treated with DMSO or 2-APB (50 µM) at 12 h post-transfection; after a further 24-h incubation, cells were stained with Fluo-4 AM and Ca^2+^ concentrations were measured. (**D**) N2a cells were cultured in Ca^2+^-free medium and transfected with Vector or CVS-M-FLAG for 36 h and then stained with Rhod-2 AM, and Ca^2+^ concentrations were measured. The fluorescence signals of Fluo-4 AM were acquired under 552 nm excitation light, quantified as the ratio of (Ca^2+^ concentration)/(Ca^2+^ concentration of control group) × 100%. (**E**) N2a cells were transfected with Vector or CVS-M for 36 h and then were processed and observed for the morphology of the ER via electron microscopy. White boxes indicate the enlarged sections of images. ER is surrounded by white dotted lines. Scale bar, 1 µm. (**F**) Ds-red ER was co-expressed with the Vector, FLAG-CVS-N, CVS-P-FLAG, CVS-G-FLAG, or CVS-M-FLAG for 36 h. Then, the cells were observed for the morphology of ER by confocal microscopy. Scale bar, 5 µm. (**G**) N2a cells were mock-infected or infected with CVS (MOI = 1) for 36 h and then stained with Fluo-4 AM, and Ca^2+^ concentrations were measured. (**H**) N2a cells were transfected with Ds-red ER plasmids for 12 h and then were mock-infected or infected with rCVS-GFP-P (MOI = 1) for 36 h; the living cells were observed for the morphology of ER by confocal microscopy. Scale bar, 5 µm. (**I**) N2a cells were transfected with Ds-red ER plasmids for 12 h and then were mock-infected or infected with rCVS-GFP-P (MOI = 1) for 36 h. Fluorescence recovery after photobleaching (FRAP) experiments were performed on areas of white boxes located inside compartments. Recovery fluorescence was measured every 500 ms for 19 s. Infected cells before photobleaching (left) and zoomed-in pictures taken at indicated times after photobleaching (right). Squares represent the zoomed-in sections. FRAP data were corrected for background, normalized to the minimum and maximum intensities. The mean is shown in black line with red/blue zone representing the SD (red line: Mock; blue line: rCVS-GFP-P). Statistical comparison of the two data sets: Kolmogorov Smirnov test, *P* < 0.0001. Scale bar, 10 µm. Error bars, mean ± SD of three experiments. Statistical analysis of comparisons between groups was carried out by Student’s *t*-test (*P < 0.05, **P < 0.01, and ***P < 0.001; ns, not significant). See also Fig. S2.

Previous studies have reported that reductions in NAD^+^ levels can trigger the release of Ca^2+^ from intracellular stores in neurons (25). Consistent with this, we observed that expression of CVS-M-FLAG increased intracellular Ca^2+^ concentrations (Fig. 2A). To exclude the contribution of extracellular Ca^2+^, we cultured N2a cells in Ca^2+^-free medium and expressed CVS-M-FLAG, which also resulted in increased intracellular Ca^2+^ concentrations (Fig. 2B), confirming the release of Ca^2+^ from intracellular stores. We also measured and confirmed increased intracellular Ca^2+^ concentrations following transfection with Slc25a4 siRNA (Fig. S2A).

Intracellular Ca^2+^ stores include the endoplasmic reticulum and mitochondria, so we sought to determine whether CVS-M induced Ca^2+^ release from both organelles. We treated CVS-M-FLAG-expressing cells with 2-APB, an inhibitor of endoplasmic reticulum Ca^2+^ channels, and found that 2-APB treatment did not fully reverse the increase in Ca^2+^ concentration induced by CVS-M-FLAG expression (Fig. 2C). Using the Rhod-2 AM probe to measure mitochondrial Ca^2+^ concentrations, we observed a significant decrease in mitochondrial Ca^2+^ levels following CVS-M-FLAG expression (Fig. 2D). These results suggest that CVS-M induces the release of Ca^2+^ from intracellular stores.

Excessive release of Ca^2+^ can result in morphological alterations in intracellular Ca^2+^ stores (28). To investigate this, we performed transmission electron microscopy (TEM) to examine the morphology of the endoplasmic reticulum and mitochondria following CVS-M expression. Our results showed no changes in mitochondrial morphology, but the ER appeared swollen and shortened in length (Fig. 2E). We also introduced a Ds-red ER plasmid, which can stain the ER in living cells. The confocal images showed that the ER morphology was altered and showed punctate aggregation in CVS-M-FLAG-expressing N2a cells but not in FLAG-CVS-N-, CVS-P-FLAG-, or CVS-G-FLAG-expressing cells (Fig. 2F). In addition, we stained a marker protein for ER, calnexin, to test whether CVS-M-FLAG did change the ER morphology. As expected, the confocal images showed that only CVS-M-FLAG could cause the altered ER morphology (Fig. S2B).

As morphological changes in the endoplasmic reticulum have been linked to the activation of unfolded protein response (UPR) (29), we also tested whether any of the CVS viral structural proteins could induce UPR, and the quantitative Real-Time PCR (qPCR) results showed that none of the structural proteins could induce UPR (Fig. S2C to S2F), confirming that morphological changes in the endoplasmic reticulum induced by CVS-M expression are not associated with UPR.

CVS infection also increased intracellular Ca^2+^ concentrations (Fig. 2G). So, we used confocal microscopy and FRAP to assess the morphology and dynamics of the endoplasmic reticulum following CVS infection in living cells. Confocal images revealed the formation of dot-like structures in the endoplasmic reticulum upon CVS infection (Fig. 2H), while FRAP analysis showed significantly reduced mobility of the endoplasmic reticulum (Fig. 2I). Collectively, these results demonstrate that CVS-M expression increases intracellular Ca^2+^ concentrations by releasing Ca^2+^ from intracellular stores and induces morphological changes in the endoplasmic reticulum.

### Excessive intracellular Ca^2+^ concentration leads to microtubule degradation in N2a cells

**Fig 3:**
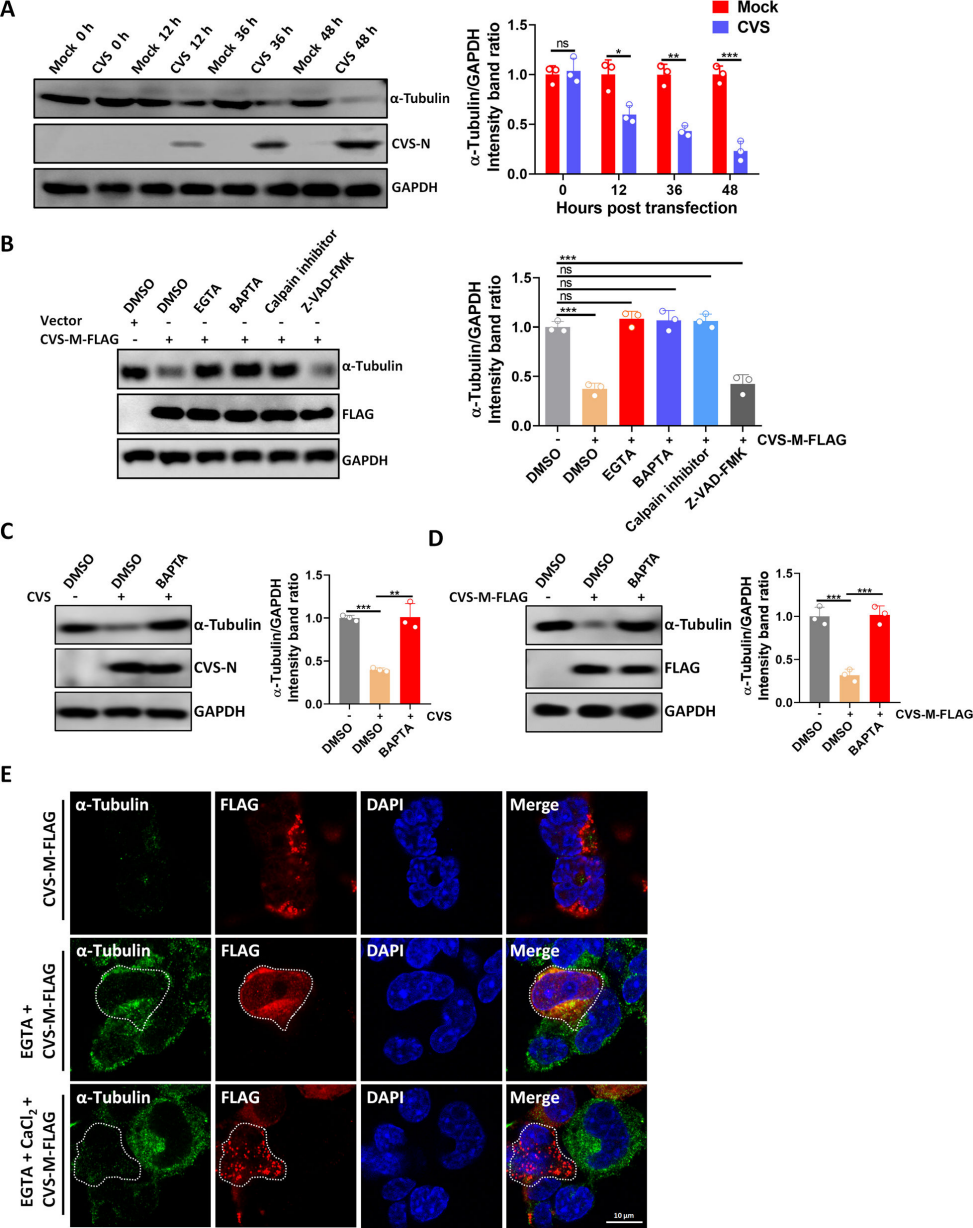
Excessive intracellular Ca^2+^ concentration activates calpain to degrade the microtubule of N2a cells. (**A**) N2a cells were mock infected or infected with CVS (MOI = 1) for indicated hours. Lysates were analyzed by WB. (**B**) N2a cells were transfected with Vector or CVS-M-FLAG for 12 h; then, cells were treated with DMSO, EGTA-AM (1 mM), BAPTA-AM (25 µM), Calpain inhibitor III (20 µM), or Z-VAD-FMK (20 µM) for 24 h; lysates were analyzed by WB. (**C**) SK-N-SH cells were mock infected or infected with CVS (MOI = 1) for 36 h or infected with CVS (MOI = 1) for 12 h and treated with BAPTA-AM (25 µM) for 24 h; lysates were analyzed by WB. (**D**) SK-N-SH cells were transfected with Vector or CVS-M-FLAG for 36 h or transfected with CVS-M-FLAG for 12 h and treated with BAPTA-AM (25 µM) for 24 h; lysates were analyzed by WB. (**E**) N2a cells were transfected with CVS-M-FLAG for 12 h; then, cells were treated with DMSO, EGTA-AM (1 mM), or EGTA-AM (1 mM) + CaCl_2_ (2 mM) for 24 h; cells were fixed and observed for α-tubulin by confocal microscopy. Cells to be observed are indicated by dashed lines. Scale bar, 10 µm. Error bars, mean ± SD of three experiments. Statistical analysis of comparisons between groups was carried out by Student’s *t*-test (*P < 0.05, **P < 0.01, and ***P < 0.001; ns, not significant). Western blot data are representative of three independent experiments. See also Fig. S3.

Elevated Ca^2+^ concentrations in neuronal axons can activate Ca^2+^-dependent proteinase calpains, leading to the degradation of cytoskeletal proteins such as tubulin and spectrin (30, 31). The α-tubulin protein level under CVS infection was then measured; the WB results showed that α-tubulin level decreased continuously with the prolongation of infection time (Fig. 3A).

Previous studies have reported that treatment with the Ca^2+^ chelator EGTA or a calpain inhibitor can partially protect axons from degradation following RABV infection. We tested whether CVS-M-induced degradation of α-tubulin was mediated by increased intracellular Ca^2+^ concentrations. As lyssavirus M protein has been reported to induce apoptosis via a TRAIL-dependent mechanism requiring the activation of caspase-8 and caspase 9 (32, 33), we investigated whether apoptosis induction was associated with CVS-M-induced α-tubulin degradation using the pan caspase inhibitor Z-VAD-FMK. Our results showed that treatment with EGTA-AM, BAPTA-AM (a cell-permeable Ca^2+^ chelator), or a calpain inhibitor inhibited CVS-M-induced degradation of α-tubulin, while inhibition of apoptosis had no effect (Fig. 3B). Correspondingly, increasing Slc25a4 can rescue the degradation of α-tubulin caused by CVS-M overexpression (Fig. S3A).

Since the amino acid sequences between human and mouse Slc25a4 are almost identical (Fig. S3B), we used the human neuroblastoma cell line SK-N-SH and showed that both CVS infection and CVS-M overexpression could lead to the decrease of α-tubulin levels in SK-N-SH cells. And the treatment with BAPTA-AM can restore this phenotype (Fig. 3C and D).

In addition, we used confocal analysis and showed that after EGTA treatment of the cells, the additional addition of Ca^2+^ enabled the degradation of α-tubulin by CVS-M (Fig. 3E). Taken together, these results suggest that CVS-M-induced degradation of α-tubulin is mediated by calpain activation following increased intracellular Ca^2+^ concentrations.

### The matrix protein of the RABV DRV-Mexico strain does not degrade α-tubulin

**Fig 4:**
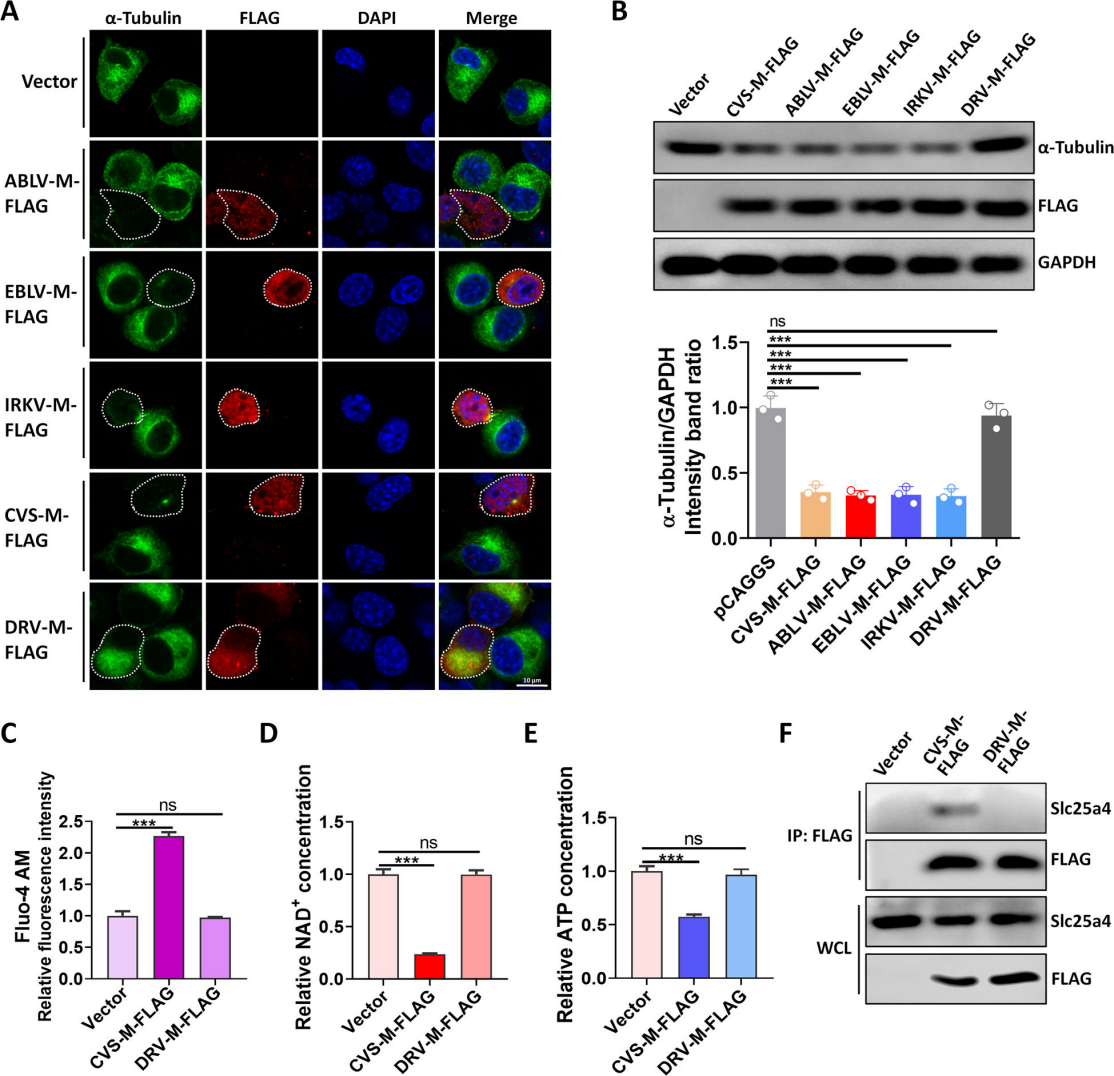
DRV-Mexico strain matrix protein does not disrupt mitochondrial metabolism. (**A**) N2a cells were transfected with Vector, ABLV-M-FLAG, EBLV-M-FLAG, IRKV-M-FLAG, CVS-M-FLAG, or DRV-M-FLAG for 36 h; cells were fixed and observed for α-tubulin by confocal microscopy. Cells to be observed are indicated by dashed lines. Scale bar, 10 µm. (**B**) N2a cells were transfected with Vector, ABLV-M-FLAG, EBLV-M-FLAG, IRKV-M-FLAG, CVS-M-FLAG, or DRV-M-FLAG for 36 h; lysates were analyzed by WB. (**C**) N2a cells were transfected with Vector, CVS-M-FLAG, or DRV-M-FLAG for 36 h and then stained with Fluo-4 AM, and Ca^2+^ concentrations were measured. (**D, E**) N2a cells were transfected with Vector, CVS-M-FLAG, or DRV-M-FLAG for 36 h; the levels of NAD^+^ (**D**) and ATP (**E**) were measured. (**F**) N2a cells were transfected with Vector, CVS-M-FLAG, or DRV-M-FLAG for 36 h; cell lysates were subjected to Co-IP and analyzed by WB. Error bars, mean ± SD of three experiments. Statistical analysis of comparisons between groups was carried out by Student’s *t*-test (*P < 0.05, **P < 0.01, and ***P < 0.001; ns, not significant). Western blot data are representative of three independent experiments.

To extend our findings to other lyssaviruses, we selected M proteins from Australian bat lyssavirus (ABLV), European bat lyssavirus 1 (EBLV-1), Bokeloh bat lyssavirus (BBLV), and a dog RABV strain from Mexico (DRV-Mexico) and generated pCAGGS-ABLV-M-FLAG (named ABLV-M-FLAG), pCAGGS-EBLV-M-FLAG (named EBLV-M-FLAG), pCAGGS-BBLV-M-FLAG (named BBLV-M-FLAG), and pCAGGS-DRV-M-FLAG (named DRV-M-FLAG) expression plasmids. Following the transfection of these plasmids into N2a cells, we observed that DRV-M-FLAG did not degrade α-tubulin (Fig. 4A and B).

To investigate why DRV-M-FLAG did not degrade α-tubulin, we measured intracellular Ca^2+^ concentrations in DRV-M-FLAG-expressing cells. Our results showed that CVS-M-FLAG increased intracellular Ca^2+^ concentrations, while DRV-M-FLAG did not (Fig. 4C). Further experiments showed that DRV-M-FLAG did not reduce cellular ATP or NAD^+^ levels like CVS-M-FLAG (Fig. 4D and E) and lost the ability to interact with Slc25a4 (Fig. 4F). Collectively, these results suggest that the M protein from DRV-Mexico does not degrade α-tubulin because it does not interact with Slc25a4 to inhibit ATP and NAD^+^ production.

### The 57th amino acid of CVS-M is responsible for α-tubulin degradation

**Fig 5:**
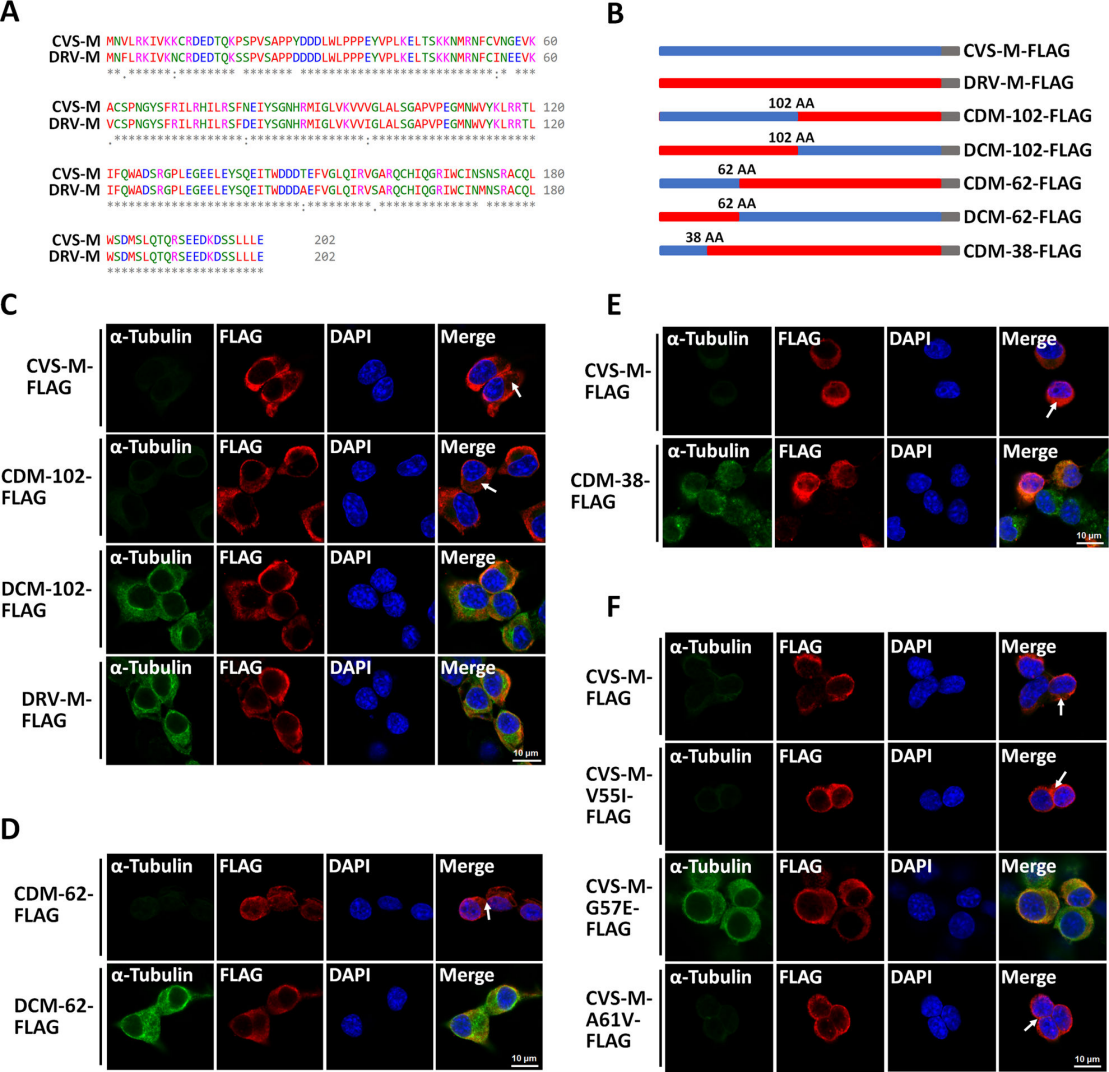
The 57th amino acid of CVS-M is the key site for α-tubulin degradation . (**A**) The amino acid sequences of CVS-M and DRV-M were compared using Clustal Omega (https://www.ebi.ac.uk/Tools/msa/clustalo/). (**B**) Schematic diagram of the construction of different M-FLAG mutants. (**C**) N2a cells were transfected with CVS-M-FLAG, CDM-102-FLAG, DCM-102-FLAG, or DRV-M-FLAG for 36 h; cells were fixed and observed for α-tubulin by confocal microscopy. White arrows indicate cells with low α-tubulin signals. Scale bar, 10 µm. (**D**) N2a cells were transfected with CDM-62-FLAG or DCM-62-FLAG for 36 h; cells were fixed and observed for α-tubulin by confocal microscopy. White arrows indicate cells with low α-tubulin signals. Scale bar, 10 µm. (**E**) N2a cells were transfected with CVS-M-FLAG or CDM-38-FLAG for 36 h; cells were fixed and observed for α-tubulin by confocal microscopy. White arrows indicate cells with low α-tubulin signals. Scale bar, 10 µm. (**F**) N2a cells were transfected with CVS-M-FLAG, CVS-M-V55I-FLAG, CVS-M-G57E-FLAG, or CVS-M-A61V-FLAG for 36 h; cells were fixed and observed for α-tubulin by confocal microscopy. White arrows indicate cells with low α-tubulin signals. Scale bar, 10 µm.

After observing that DRV-M-FLAG did not induce α-tubulin degradation or other phenotypes, we sought to identify the key amino acids in CVS-M-FLAG responsible for α-tubulin degradation by generating mutants. Following a comparison of the amino acid sequences of the two proteins using Clustal Omega, an online multiple sequence alignment tool (34) (Fig. 5A), we generated pCAGGS-FLAG expression plasmids encoding DRV-M with the first 102 amino acids replaced by those of CVS-M (named CDM-102-FLAG) and CVS-M with the first 102 amino acids replaced by those of DRV-M (named DCM-102-FLAG). Our results showed that CDM-102-FLAG induced cytoskeletal degradation while DCM-102-FLAG did not, suggesting that the key amino acids in CVS-M-FLAG responsible for α-tubulin degradation are located within the first 102 amino acids (Fig. 5B and C).

We then generated pCAGGS-FLAG expression plasmids encoding DRV-M with the first 62 amino acids replaced by those of CVS-M (named CDM-62-FLAG) and CVS-M with the first 62 amino acids replaced by those of DRV-M (named DCM-62-FLAG). Our results showed that CDM-62-FLAG induced α-tubulin degradation while DCM-62-FLAG did not (Fig. 5D), indicating that the key amino acids in CVS-M-FLAG responsible for α-tubulin degradation are located within the first 62 amino acids. We further generated a pCAGGS-FLAG expression plasmid encoding DRV-M with the first 38 amino acids replaced by those of CVS-M (named CDM-38-FLAG) and found that CDM-38-FLAG did not induce α-tubulin degradation (Fig. 5E), suggesting that the key amino acids in CVS-M-FLAG responsible for α-tubulin degradation are located between amino acids 38 and 62.

The amino acid sequences of the two M proteins differed at three positions between amino acids 38 and 62: positions 55, 57, and 61. We mutated these amino acids in CVS-M to their corresponding residues in DRV-M and generated a series of pCAGGS-FLAG expression plasmids (named CVS-M-V55I-FLAG, CVS-M-G57E-FLAG, and CVS-M-A61V-FLAG). Our results showed that mutating glycine at position 57 in CVS-M to glutamate prevented CVS-M from degrading α-tubulin (Fig. 5F). Collectively, these findings confirm that the key residue for CVS-M-induced α-tubulin degradation is the 57th amino acid.

### The M protein of several lyssaviruses carrying the 57th amino acid mutation does not degrade α-tubulin

**Fig 6:**
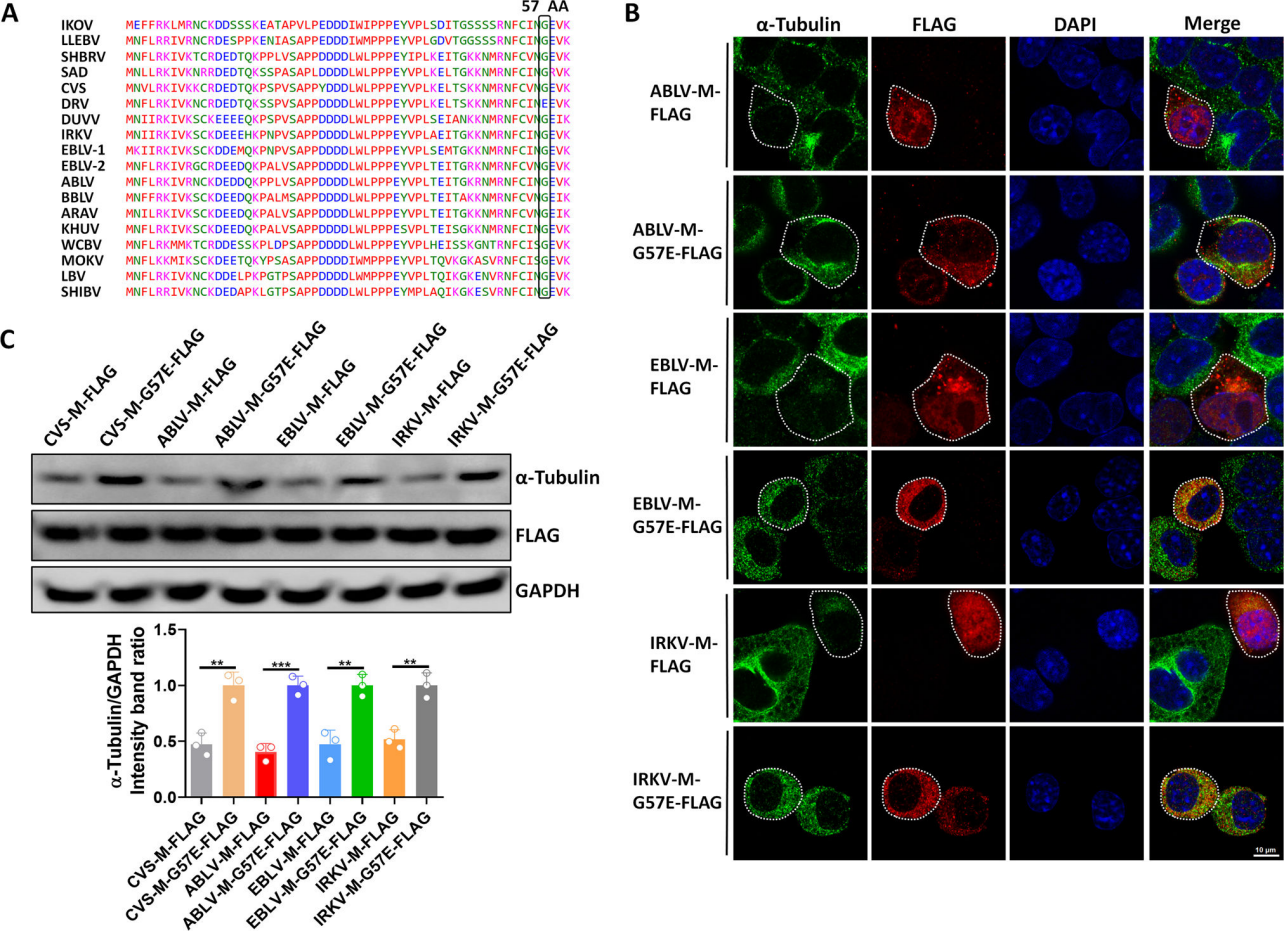
The 57th amino acid of the lyssavirus matrix protein is essential for α-tubulin degradation. (**A**) The N-terminal amino acid sequences of several typical lyssavirus strains were compared using Clustal Omega. The 57th amino acid was marked. IKOV, Ikoma lyssavirus; LLEBV, Lleida bat lyssavirus; SHBRV, rabies virus (strain SHBRV-18); SAD, rabies virus (strain SAD-B19); CVS, rabies virus (strain CVS-11); DRV, rabies virus (strain DRV-Mexico); DUVV, Duvenhage virus; IRKV, Irkut virus; EBLV-1, European bat lyssavirus 1; EBLV-2, European bat lyssavirus 2; ABLV, Australian bat lyssavirus; BBLV, Bokeloh bat lyssavirus; ARAV, Aravan virus; KHUV, Khujand lyssavirus; WCBV, West Caucasian bat virus; MOKV, Mokola virus; LBV, Lagos bat virus; SHIBV, Shimoni bat virus. (**B**) N2a cells were transfected with ABLV-M-FLAG, ABLV-M-G57E-FLAG, EBLV-M-FLAG, EBLV-M-G57E-FLAG, IRKV-M-FLAG, or IRKV-M-G57E-FLAG for 36 h; cells were fixed and observed for α-tubulin by confocal microscopy. Cells to be observed are indicated by dashed lines. Scale bar, 10 µm. (**C**) N2a cells were transfected with ABLV-M-FLAG, ABLV-M-G57E-FLAG, EBLV-M-FLAG, EBLV-M-G57E-FLAG, IRKV-M-FLAG, or IRKV-M-G57E-FLAG for 36 h; lysates were analyzed by WB. Error bars, mean ± SD of three experiments. Statistical analysis of comparisons between groups was carried out by Student’s *t*-test (*P < 0.05, **P < 0.01, and ***P < 0.001; ns, not significant). Western blot data are representative of three independent experiments.

To assess the conservation of amino acid 57 in lyssaviruses, we compared the sequences of M proteins from representative lyssavirus strains and found that amino acid 57 is conserved in all strains except DRV-Mexico (Fig. 6A).

We then mutated amino acid 57 in the M proteins of ABLV, EBLV-1, and BBLV and generated pCAGGS-FLAG expression plasmids encoding these mutants (named ABLV-M-G57E-FLAG, EBLV-M-G57E-FLAG, and BBLV-M-G57E-FLAG). Following transfection of these plasmids into N2a cells, we observed that ABLV-M-G57E-FLAG, EBLV-M-G57E-FLAG, and BBLV-M-G57E-FLAG lost their ability to degrade α-tubulin (Fig. 6B and C). These results confirm that the 57th amino acid of lyssavirus M proteins is critical for M-induced α-tubulin degradation.

### CVS-M-G57E does not disrupt mitochondrial metabolism

**Fig 7:**
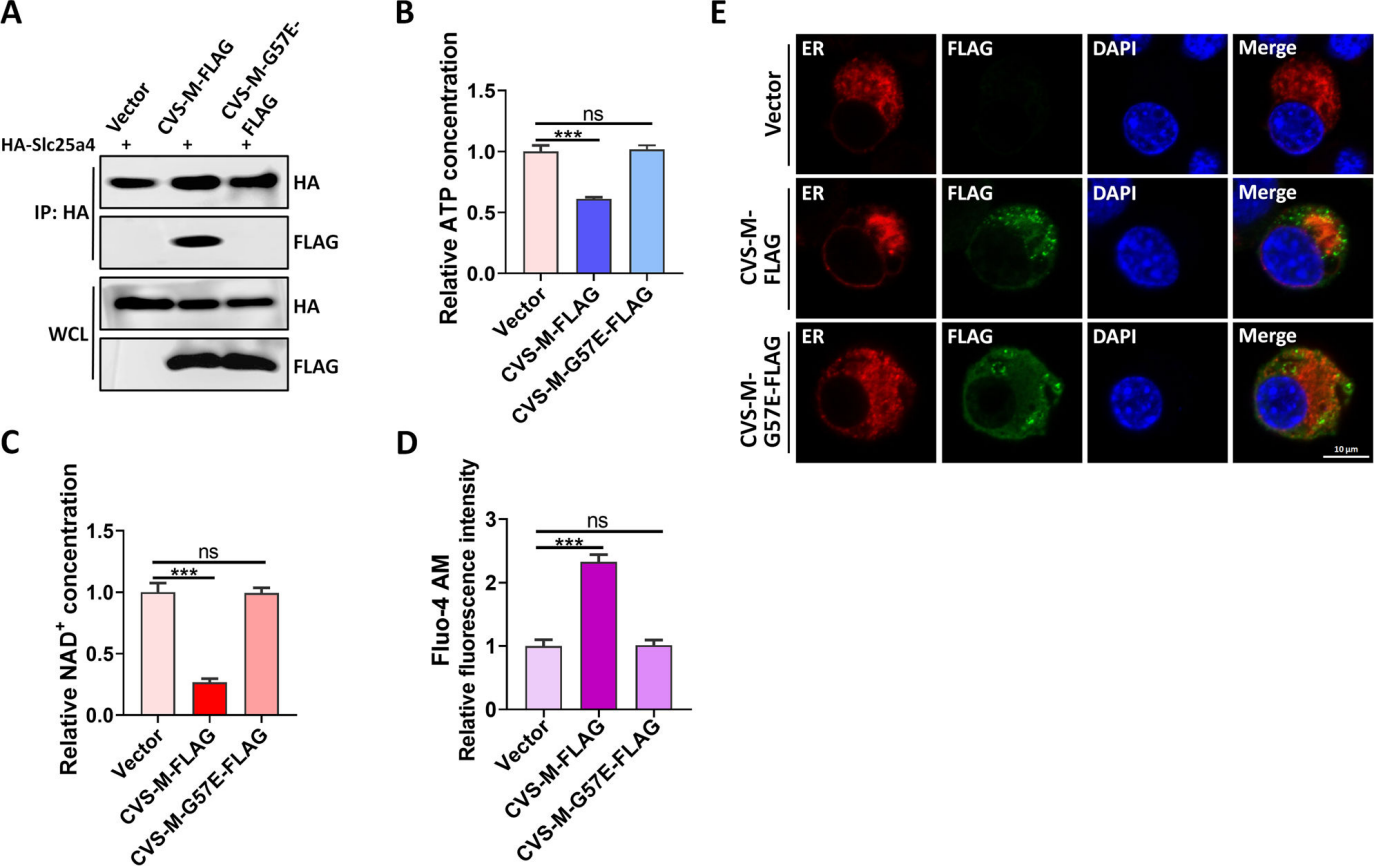
Mitochondrial metabolism is not disturbed by CVS-M-G57E. (**A**) N2a cells were transfected with Vector, CVS-M-FLAG, or CVS-M-G57E-FLAG for 36 h; cell lysates were subjected to Co-IP and analyzed by WB. (**B, C**) N2a cells were transfected with Vector, CVS-M-FLAG, or CVS-M-G57E-FLAG for 36 h; the levels of ATP (**B**) and NAD^+^ (**C**) were measured. (**D**) N2a cells were transfected with Vector, CVS-M-FLAG, or CVS-M-G57E-FLAG for 36 h and then stained with Fluo-4 AM, and Ca^2+^ concentrations were measured. (**E**) Ds-red ER was co-expressed with the expression vector, CVS-M-FLAG, or CVS-M-G57E-FLAG for 36 h and then fixed and stained to observe ER morphology. Scale bar, 10 µm. Error bars, mean ± SD of three experiments. Statistical analysis of comparisons between groups was carried out by Student’s *t*-test (*P < 0.05, **P < 0.01, and ***P < 0.001; ns, not significant). Western blot data are representative of three independent experiments.

After confirming that the 57th amino acid of lyssavirus M proteins is critical for M-induced α-tubulin degradation, we sought to elucidate the underlying mechanism. Co-IP experiments confirmed that CVS-M-FLAG interacted with HA-Slc25a4, while CVS-M-G57E-FLAG did not (Fig. 7A). Furthermore, CVS-M-G57E-FLAG failed to reduce cellular ATP and NAD^+^ levels (Fig. 7B and C) or increase intracellular Ca^2+^ concentrations (Fig. 7D). Confocal imaging also showed that CVS-M-G57E-FLAG expression did not alter the morphology of the endoplasmic reticulum (Fig. 7E). These results are highly consistent with those obtained using DRV-M-FLAG and further support our conclusion that CVS-M-G57E-FLAG does not degrade α-tubulin because it does not interact with Slc25a4.

### The recombinant RABV CVS-M-G57E induces fewer neuronal degenerative changes

**Fig 8:**
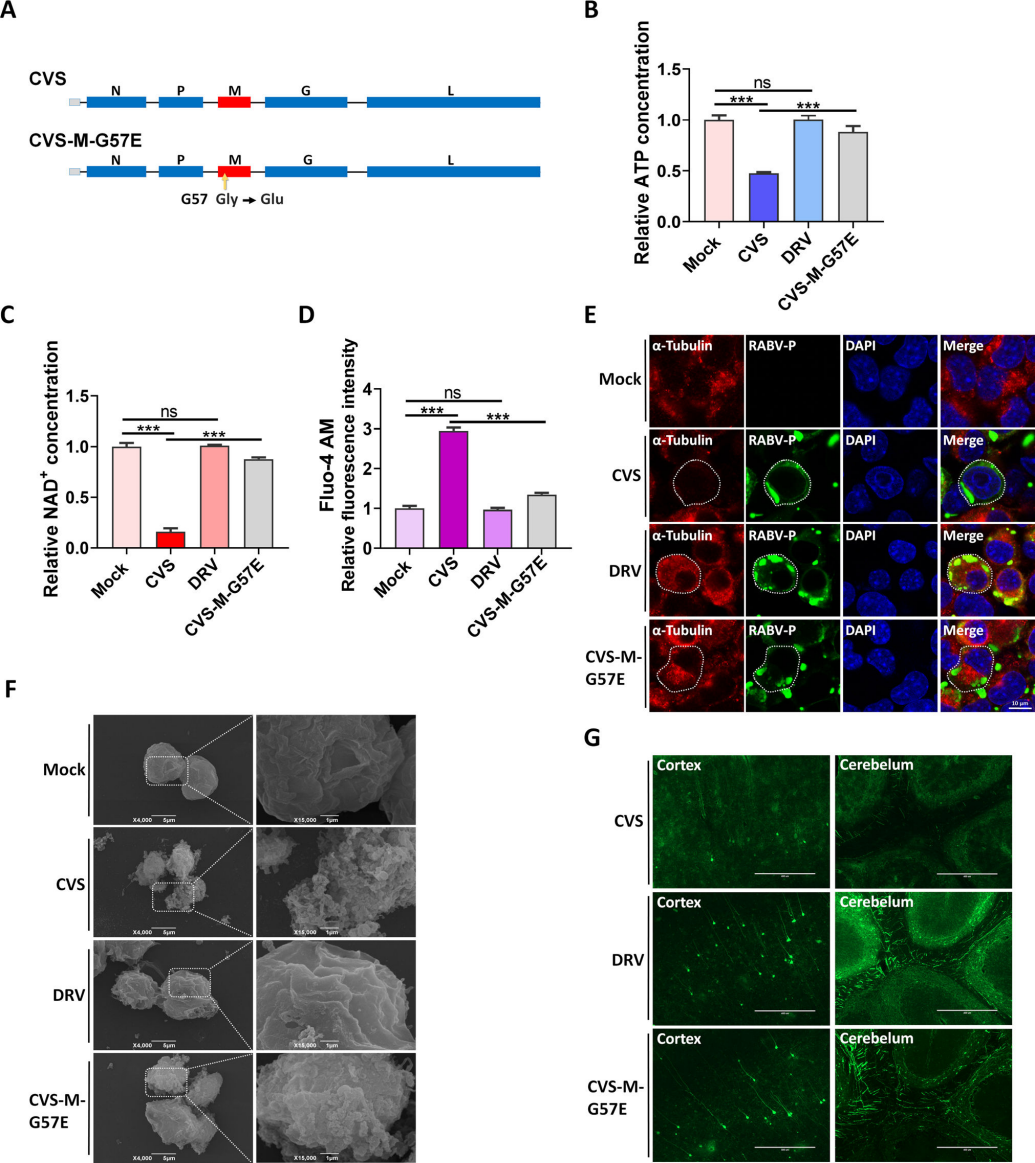
Less neuronal degenerative changes are induced by the recombinant rabies virus CVS-M-G57E. (**A**) Schematic diagram of the construction of two recombinant rabies viruses: CVS and CVS-M-G57E. (**B, C**) N2a cells were mock-infected or infected with CVS (MOI = 1), DRV (MOI = 10), and CVS-M-G57E (MOI = 10) for 36 h; the levels of ATP (**B**) and NAD^+^ (**C**) were measured. (**D**) N2a cells were mock infected or infected with CVS (MOI = 1), DRV (MOI = 10), and CVS-M-G57E (MOI = 10) for 36 h and then stained with Fluo-4 AM, and Ca^2+^ concentrations were measured. (**E**) N2a cells were mock infected or infected with CVS (MOI = 1), DRV (MOI = 10), and CVS-M-G57E (MOI = 10) for 36 h; cells were fixed and observed for α-tubulin by confocal microscopy. Cells to be observed are indicated by dashed lines. Scale bar, 10 µm. (**F**) N2a cells were mock infected or infected with CVS (MOI = 1), DRV (MOI = 10), and CVS-M-G57E (MOI = 10) for 36 h; cells were possessed and observed for the cell morphology via electron microscopy. White boxes indicate the enlarged sections of images. Scale bar, 1 µm. (**G**) Female Thy1-GFP mice (6-week-old, *n* = 9) were intracranially (i.c.) inoculated with 25 µL of CVS (20 FFU), DRV (200 FFU), or CVS-M-G57E (200 FFU). At 6 days post-infection (d.p.i.), brains were harvested and sliced for the observation of neurons by fluorescence microscopy. Scale bar, 400 µm. Error bars, mean ± SD of three experiments. Statistical analysis of comparisons between groups was carried out by Student’s *t*-test (*P < 0.05, **P < 0.01, and ***P < 0.001; ns, not significant). Western blot data are representative of three independent experiments.

To validate our findings at the viral level, we generated a recombinant RABV CVS strain carrying a mutation at the 57th amino acid of CVS-M, named CVS-M-G57E (Fig. 8A). We measured ATP and NAD^+^ levels in N2a cells infected with rCVS, DRV, or CVS-M-G57E and found that infection with DRV did not reduce intracellular ATP or NAD^+^ levels (Fig. 8B and C) and a CVS-M-G57E-induced decrease in ATP and NAD^+^ levels was significantly less than the CVS-induced decrease. Consistently, infection with DRV did not increase intracellular Ca^2+^ concentrations, while the CVS-M-G57E-induced increase in Ca^2+^ concentrations was significantly less than the CVS-induced increase. (Fig. 8D). We also observed abundant α-tubulin singles in DRV- or CVS-M-G57E-infected cells but not in CVS-infected cells (Fig. 8E). As cytoskeletal degradation can result in changes in cell morphology (35), we used scanning electron microscopy (SEM) to examine N2a cells infected with CVS, DRV, or CVS-M-G57E. Our results showed that morphological changes in DRV- or CVS-M-G57E-infected cells were less pronounced than those in CVS-infected cells (Fig. 8F).

Degradation of the neuronal axon cytoskeleton is also a contributing factor to neuronal degeneration induced by RABV. To investigate the impact of the recombinant RABV CVS-M-G57E on neuronal axons in mouse brains, we infected THY1-eGFP transgenic mice with CVS or CVS-M-G57E and examined brain sections. Our results showed that morphological changes of neurons in the CVS-M-G57E-infected mouse brain were less pronounced than those in the CVS-infected mouse brain (Fig. 8G). Taken together, our results showed that the recombinant RABV CVS-M-G57E induces fewer neuronal degenerative changes than its parent strain CVS.

## DISCUSSION

**Fig 9:**
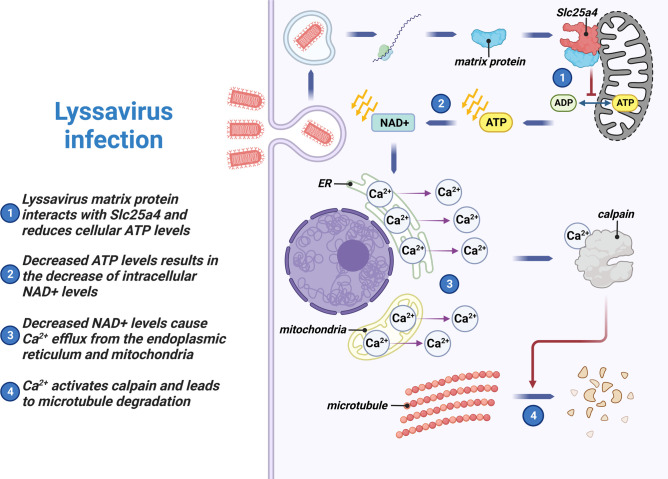
Model depicting how lyssavirus matrix protein causes microtubule degradation. Lyssavirus matrix protein was transcribed and translated after lyssavirus infection. The matrix protein interacts with Slc25a4 and disrupts mitochondrial metabolism, causing the decrease of NAD^+^ and ATP levels in neuronal cells. The reduction of NAD^+^ leads to the release of Ca^2+^ from intracellular calcium stores such as the endoplasmic reticulum and mitochondria. The high concentration of Ca^2+^ in the neuronal cytoplasm activates the Ca^2+^-dependent proteinase calpain to degrade microtubules. This figure was created with BioRender (https://www.biorender.com/).

This study discovered that the matrix protein of the RABV CVS strain inhibits the production of ATP and NAD^+^ in mitochondria by interacting with Slc25a4 on mitochondria, ultimately leading to the degradation of neuronal cytoskeletal proteins. Our results show that the decrease of NAD^+^ in neuronal cells leads to the release of Ca^2+^ from intracellular calcium stores such as the endoplasmic reticulum and mitochondria. The high concentration of Ca^2+^ in the neuronal cytoplasm activates Ca^2+^-dependent proteinase calpains to degrade cytoskeletal proteins (Fig. 9). This study also found that mutation of the 57th glycine residue of the RABV M protein to glutamine alters its secondary structure. The mutated M protein no longer interacts with Slc25a4, and the recombinant RABV with the 57th glycine residue of the M protein mutated to glutamine causes a decrease in the level of axonal self-destruction in mouse brains, indicating that the M protein is involved in the neuron degeneration induced by RABV infection.

RABV infection has been reported to cause degenerative changes in neuronal axons (3, 4). These changes are strongly related to the level of NAD^+^ in neuronal cells, the concentration of Ca^2+^, and the enzymatic activity of Ca^2+^-dependent proteinase calpains. It has been reported that in RABV-infected neurons, SARM1 causes degenerative changes in neuronal axons in a short period of time through rapid depletion of NAD^+^. However, knockout SARM1 neurons still undergo degenerative changes after RABV infection; only the time to degeneration is significantly prolonged, suggesting that SARM1 plays a role in RABV infection in accelerating axonal degeneration (4). The underlying mechanism of how RABV infection causes neurodegenerative changes is still unknown. Recently, it has been reported that both RABV infection and RABV M protein can degrade the microtubule of mouse neuronal cell line N2a (36). M protein can promote the acetylation level of α-tubulin by upregulating HDAC6 to eventually depolymerize α-tubulin. However, inhibition of HDAC6 activity cannot prevent the degradation of α-tubulin by M protein. This suggests that the main mechanism of M protein degradation of α-tubulin may be unrelated to HDAC6 (36). Based on the above literature, we speculate that the mechanism of RABV M protein degradation of microtubule is associated with low NAD^+^ levels and high Ca^2+^ concentration in neuronal cells

We hypothesize that the M protein decreases NAD^+^ levels in neuronal cells through an unknown mechanism, leading to the release of Ca^2+^ from the Ca^2+^ pool and an increase in intracellular Ca^2+^ concentration. This activates Ca^2+^-dependent proteinase calpains, which degrades skeletal protein. Our confocal images show that sequestering intracellular Ca^2+^ prevents M protein from degrading α-tubulin (Fig. 3B and E). Additionally, overexpression of the M protein causes a decrease in intracellular NAD^+^ levels and an increase in Ca^2+^ concentration (Fig. 1E and 2A). Despite M proteins co-localizing with mitochondria and causing apoptosis (33, 37), treatment with a pan apoptotic inhibitor Z-VAD-FMK did not prevent M proteins from degrading α-tubulin (Fig. 3B). This suggests that the mechanism by which M proteins cause α-tubulin degradation is not closely linked to apoptosis.

Sarm1 and Nmnat2 are critical markers in the process of axonal degeneration in neurons (38, 39). Nmnat2 generates NAD^+^ by depleting NMN and ATP in the neuronal cytosol (40, 41), while Sarm1 rapidly depletes NAD^+^ (20). Although Sarm1 promotes degenerative axonal changes in neurons following RABV infection, it does not determine their occurrence. Our experiments showed no co-localization of CVS-M with Nmnat2 in the Golgi apparatus and no interaction between CVS-M and Sarm1 (data not shown), suggesting that CVS-M does not directly regulate NAD^+^ levels in neurons by binding to Nmnat2 or Sarm1.

In our screening of whether M proteins of the lyssavirus could cause degradation of microtubule, we found that the M protein of the DRV strain of RABV did not cause α-tubulin degradation (Fig. 4A). DRV-M did not interact with Slc25a4 and did not cause a decrease in intracellular NAD^+^ levels (Fig. 4D and F). Since neither DRV-M nor CVS-M has a crystal structure, we performed secondary structure prediction and comparison using SOPMA (an improved self-optimized protein secondary structure prediction tool) (42) and found that DRV-M and CVS-M have a large secondary structure variability within amino acids 53–68, as evidenced by the longer extended strand and the positionally altered beta-turn in amino acids 53–68 of DRV-M (Fig. S4). We then performed secondary structure prediction and comparison of CVS-M with the mutation at position 57 and showed that the secondary structure of CVS-M-G57E is almost the same as that of DRV-M in amino acids 53–68 (Fig. S4). This result illustrates the structural importance of the 57th amino acid in the lyssavirus M protein. When performing experiments with recombinant RABVs, we found that the recombinant CVS-M-G57E RABV could still cause some ATP and NAD reduction, suggesting that factors other than the M protein influence the energy metabolism of neurons. We hypothesized that this was due to the G protein of CVS, since it has been reported that the G protein of non-pathogenic RABV strains (such as the CVS strain) can trigger apoptosis, whereas the G protein of pathogenic strains (such as the DRV strain) induces less or no apoptosis (43, 44), and activation of the apoptotic pathway inhibits mitochondrial metabolism (45).

Our study shows that most lyssavirus M proteins may have the ability to degrade neuronal cytoskeletal proteins. This is the first report that lyssavirus M proteins have been shown to affect ATP and NAD^+^ production, ultimately leading to the degradation of skeletal proteins by Ca^2+^-dependent proteases. Mouse experiments showed that neuronal axonal degeneration induced by mutant RABV unable to interact with Slc25a4 was reduced. Our findings advance the understanding of neurodegenerative changes caused by RABV infection. Some canine-derived street strains, such as the DRV-Mexico strain, may cause less severe neurodegenerative changes because their M proteins do not drastically affect mitochondrial function. More importantly, when using RABV as a tool for neurological studies, appropriate mutation of its M protein may reduce the toxicity of the viral tool to the nervous system.

## MATERIALS AND METHODS

### Cells, viruses, antibodies, inhibitors, and mice

N2a (mouse neuroblastoma, ATCC CCL-131) and SK-N-SH (human neuroblastoma, ATCC HTB-11TM) cells were cultured in a 37°C humidified 5% CO_2_ atmosphere, and growth media were Dulbecco’s modified Eagle’s medium (Thermo Fisher Scientific, USA) supplemented with 10% fetal bovine serum (Gibco) and 1% antibiotics (penicillin and streptomycin) (Beyotime). Lab-attenuated RABV strain CVS-B2c (derived from CVS-24 by passaging in BHK-21 cells) and wt RABV strain of DRV-Mexico (DRV) were stored in our laboratory. The recombinant RABVs were cloned from CVS-B2c, and the recombinant CVS and CVS-GFP-P were constructed as described previously (46).

The monoclonal antibodies (mAbs) against FLAG-tag (M185-3L), HA-tag (M180-3), and GAPDH (M171-3) were purchased from Medical & Biological Laboratories (MBL, Nagoya, Japan). The polyclonal antibodies (pAbs) against α-tubulin (AC007), Slc25a4 (A20842), and Calnexin (A15631) were purchased from ABclonal Technology (Wuhan, China). The pAbs against the RABV-P were prepared in our laboratory.

2-APB (GC17562), EGTA-AM (GB30177), BAPTA-AM (GC13517), Z-VAD-FMK (GC12861), and Thapsigargin (GC11482) were purchased from Glpbio (Montclair, CA, USA). Calpain inhibitor III (ab145601) was purchased from Abcam (Cambridge, MA, USA).

Six-week-old female Thy1-GFP mice were gifts from Dr. Xiangning Li at Huazhong University of Science and Technology and bred in the animal facility at Huazhong Agricultural University. All experiments involving mice were performed following the recommendations in the Guide for the Care and Use of Laboratory Animals of the Ministry of Science and Technology of China.

### Construction of expression plasmids

The full-length N, P, G, and M genes of RABV CVS strain were amplified from the total RNA extracted from CVS-infected N2a cells using the ReverTra Ace qPCR RT Master Mix (TOYOBO, FSQ-201) with Phanta Max Super-Fidelity DNA polymerase (Vazyme Biotech, P505-d1). After adding the FLAG-tag DNA sequence to their N- or C-terminus, the products were cloned into the pCAGGS (P19161, MIAOLING Biology, China) vector by using the SacI/NheI, EcoRI/KpnI, EcoRI/SacI, and EcoRI/SacI cloning sites, respectively. The cloning was performed using the ClonExpress II One Step Cloning Kit (Vazyme Biotech, C112-01, China). The primer sets used were designed by Primer 6 (PREMIER Biosoft Biolabs) (Table 1). Mouse Slc25a4 was amplified from N2a genomic cDNA using the specific primers (upstream primer 5′ATGGGGGATCAGGCTTTGAGCTTTC3′ and downstream primer 5′CACATATTTTTTGATCTCATCATACAATACCAATAC3′. The vector plasmid pCAGGS-HA (P0165, MIAOLING Biology) was digested with EcoRI/SacI, and the cloning was performed using the ClonExpress II One Step Cloning Kit (Vazyme Biotech, C112-01). The sequences of all constructs were verified by sequencing.

**TABLE 1:** Primers used for expression plasmids construction^*a*^

Primers	Sequences (5′−3′)
CVS-N-F	ATGGATGCCGACAAGATTGTG
CVS-N-R	TTATGAGTCATTCGAATACGTCTTGTT
CVS-P-F	ATGAGCAAGATCTTTGTTAATCCG
CVS-P-R	GCAGGATGTATAGCGATTCAAATC
CVS-G-F	ATGGTTCCTCAGGTTCTTTTGTTT
CVS-G-R	CAGTCTGATCTCACCTCCACTCTTAT
CVS-M-F	ATGAACGTTCTACGCAAGATAGTGA
CVS-M-R	TTCTAGAAGCAGAGAAGAGTCTTTGTCC
DRV-M-F	ATGAACTTTCTACGTAAGATAGTGAAAAAC
DRV-M-R	TTCTAAAAGCAGAGAAGAGTCTTTGTCC
ABLV-M-F	ATGAACTTTCTCCGCAAGATAGTCA
ABLV-M-R	CTCTAACAGGAGAGAACTGTTCTTGTCC
EBLV-M-F	ATGAAGATCATTCGGAAAATTGTTAA
EBLV-M-R	TTCGAGGAGCAAGGAAGTGTTC
IRKV-M-F	ATGAACATTATCCGCAAAATAGTGA
IRKV-M-R	TTCAAGAAGCAGGGAAGTGTTCT
pCAGGS-N-FLAG-CVS-N-F	tttggcaaagaattcgagctcGATTACAAGGATGACGACGATAAGATGGATGCCGACAAGATTGTG
pCAGGS-N-FLAG-CVS-N-R	gagggaaaaagatctgctagcTTATGAGTCATTCGAATACGTCTTGTT
pCAGGS-C-FLAG-CVS-P-F	catcattttggcaaagaattcATGAGCAAGATCTTTGTTAATCCG
pCAGGS-C-FLAG-CVS-P-R	ctcgaggcatgcccgggtaccTTACTTATCGTCGTCATCCTTGTAATCGCAGGATGTATAGCGATTCAAATC
pCAGGS-C-FLAG-CVS-G-F	catcattttggcaaagaattcATGGTTCCTCAGGTTCTTTTGTTT
pCAGGS-C-FLAG-CVS-G-R	gtaccatgcatcgatgagctcTTACTTATCGTCGTCATCCTTGTAATCCAGTCTGATCTCACCTCCACTCTTAT
pCAGGS-C-FLAG-CVS-M-F	catcattttggcaaagaattcATGAACGTTCTACGCAAGATAGTGA
pCAGGS-C-FLAG-CVS-M-R	gtaccatgcatcgatgagctcTTACTTATCGTCGTCATCCTTGTAATCTTCTAGAAGCAGAGAAGAGTCTTTGTCC
pCAGGS-C-FLAG-DRV-M-F	catcattttggcaaagaattcATGAACTTTCTACGTAAGATAGTGAAAAAC
pCAGGS-C-FLAG-DRV-M-R	gtaccatgcatcgatgagctcTTACTTATCGTCGTCATCCTTGTAATCTTCTAAAAGCAGAGAAGAGTCTTTGTCC
pCAGGS-C-FLAG-ABLV-M-F	catcattttggcaaagaattcATGAACTTTCTCCGCAAGATAGTCA
pCAGGS-C-FLAG-ABLV-M-R	gtaccatgcatcgatgagctcTTACTTATCGTCGTCATCCTTGTAATCCTCTAACAGGAGAGAACTGTTCTTGTCC
pCAGGS-C-FLAG-EBLV-M-F	catcattttggcaaagaattcATGAAGATCATTCGGAAAATTGTTAA
pCAGGS-C-FLAG-EBLV-M-R	gtaccatgcatcgatgagctcTTACTTATCGTCGTCATCCTTGTAATCTTCGAGGAGCAAGGAAGTGTTC
pCAGGS-C-FLAG-IRKV-M-F	catcattttggcaaagaattcATGAACATTATCCGCAAAATAGTGA
pCAGGS-C-FLAG-IRKV-M-R	gtaccatgcatcgatgagctcTTACTTATCGTCGTCATCCTTGTAATCTTCAAGAAGCAGGGAAGTGTTCT

^*a*^ Lowercase letters represent recombinant homology arm sequences.

### NAD^+^ measurement

A commercially available NAD+/NADH Quantification Kit (S0175, Beyotime) was used to measure cellular NAD+ levels. Briefly, 1 × 10^6^ N2a cells of each group were collected and lysed in 200 µL of NAD^+^/NADH extraction buffer for 30 min on ice. Whole-cell extracts were then clarified by centrifugation for 10 min at 12,000 *g*. The suspension was used to measure total NAD^+^/NADH. Half of the cell extract was transferred to a new tube and incubated at 60°C for 30 min to decompose the NAD^+^ while NADH was left. Total NAD^+^/NADH or NADH samples were added to a 96-well plate at 20 µL/well. Subsequently, 90 µL of alcohol dehydrogenase solution was added and incubated at 37°C for 10 min. Finally, 10 µL of chromogenic solution was added to each well and the mixture was incubated at 37°C for 30 min. The absorbance values were measured at 450 nm. The amount of NAD^+^ was calculated by subtracting NADH from the total NAD^+^/NADH.

### ATP measurement

A commercially available ATP Quantification Kit (S0026, Beyotime) was used to measure cellular ATP levels. Briefly, the cultured N2a cells to be tested were removed from the culture solution and then lysed in 200 µL of lysis buffer for 30 min on ice. Whole-cell extracts were clarified by centrifugation for 10 min at 12,000 *g*. The suspension was used to measure ATP. Add 100 µL of ATP assay working solution to the assay wells, then add 20 µL of the sample to the assay wells and mix, and then detect by a multi-function measuring instrument: Spark 10M (PE, USA).

### Confocal microscopy

N2a cells were washed three times with PBS and fixed with 4% paraformaldehyde for 30 min at room temperature and then washed three times with PBS. Then, the cells were permeabilized with 0.1% Triton X-100 and then blocked with 10% goat serum (C0265, Beyotime) which were diluted with PBS for 2 h at 37°C and probed with anti-FLAG antibodies (M185-3L, MBL) and anti-α-tubulin antibodies (AC007, ABclonal) which were diluted with PBS and 5% (wt/vol) BSA for 12 h at 4°C, treated with Alexa FluorTM 488 goat anti-rabbit IgG (A11034, Invitrogen) and goat anti-mouse IgG DylightTM 594 conjugated antibodies (35511, Invitrogen) as secondary antibodies for 1 h at room temperature. Nuclei were stained with 4,6-diamidino-2-phenylindole (DAPI) (C1002, Beyotime) for 10 min at room temperature. Cells were again washed three times with PBS and then imaged with a Zeiss LSM 880 confocal microscope under an oil objective (Carl Zeiss AG, Oberkochen, Germany). A resolution of 1,024 × 1,024 pixels and an average number of two pictures were utilized to acquire all confocal images.

For observing ER in living cells, N2a cells were transfected with pDsRed2-ER plasmids. For staining of nuclei in living cells, N2a cells were treated with Hoechst 33342 (C1022, Beyotime). For staining of mitochondria (Mito) in cells, N2a cells were treated with Mito-Tracker Red CMXRos (C1049B, Beyotime). For staining of rabies virus phosphoprotein (RABV-P) in cells, N2a cells were probed with anti-RABV-P antibodies (prepared in our lab).

### Immunoprecipitation/Mass Spectrometry

N2a cells were transfected with CVS-M-FLAG for 36 h. Cells were harvested and lysed via lysis buffer for 30 min at 4°C. The supernatants were collected via centrifugation at 10,000 rpm for 30 min at 4°C and coimmunoprecipitated with immunoglobulin G (IgG) or anti-FLAG (M185-3L, MBL). The immunoprecipitated fractions were separated via 10% SDS-PAGE and analyzed via silver staining. Multiple electrophoretic bands were cut and analyzed via mass spectrometry. The mass spectrometry proteomics data have been deposited to the ProteomeXchange Consortium (http://proteomecentral.proteomexchange.org) via the iProX partner repository (47, 48) with the data set identifier PXD044493.

### Co-immunoprecipitation

For the Co-IP assay, N2a cells were cotransfected with the mammalian expression vector pCAGGS expressing different target proteins. At 36 h post-transfection, the cells were lysed in RIPA buffer (P0013, Beyotime) supplemented with a protease inhibitor cocktail (Roche) for 30 min on ice. The cell lysates were centrifuged for 10 min at 12,000 rpm and 4°C, and the supernatants were transferred into a new tube and pretreated with rProtein A/G MagPoly Beads (SM01505, Smart-Lifesciences, China) for 1 h at 4°C to remove non-specific binding proteins from magnetic beads. Pretreated supernatants were further incubated with mAbs against FLAG or HA overnight at 4°C, and then, fresh magnetic beads washed with PBS were added and incubated for 3 h at 4°C with rotation. The samples were then washed five times with ice-cold PBS, and the bound proteins were eluted by boiling in 4× SDS-PAGE loading buffer and analyzed by Western blotting with the indicated antibodies.

### Mitochondria isolation

Mitochondrial isolation from N2a cells was performed by using a commercial mitochondria isolation kit (C3601, Beyotime) as per the manufacturer’s protocol.

### Calcium-ion fluorescent probe

The Ca^2+^ testing of N2a cells was performed using a Fluo-4 AM (S1060, Beyotime) or a Rhod-2 AM (ab142780, Abcam) according to the manufacturer’s instructions. Briefly, the cultured cells to be tested were removed from the culture solution, and then, Fluo-4 AM working fluid was added. The sample was then incubated with a fluorescent probe and then reincubated for 30 min to stimulate the cells. The fluorescence of Fluo-4 was detected by a multi-function measuring instrument: Spark 10M (PE, USA).

### Western blotting

N2a cells under different treatments were lysed in RIPA buffer (P0013, Beyotime) supplemented with a protease inhibitor cocktail (Roche) for 30 min on ice. The total cell lysates were separated on 12%–14% SDS-PAGE gels and transferred to PVDF membranes (Bio-Rad). Membranes were blocked with TBST with 5% (wt/vol) non-fat dry milk for 4 h and probed with primary antibodies which were diluted with TBST and 5% (wt/vol) non-fat dry milk overnight at 4°C. After rinsing, membranes were probed with HRP-conjugated goat anti-mouse (BA1051, Boster, Wuhan, China) or goat anti-rabbit secondary antibodies (BA1055, Boster) and then developed using the BeyoECL Star Kit (P0018A, Beyotime). Images were captured with an Amersham Imager 600 (GE Healthcare) imaging system. Three independent biological experiments were performed for western blotting quantification analysis. ImageJ software (National Institutes of Health, Bethesda, MD, USA) was used to quantify the intensity of protein bands.

### siRNAs

Mouse Slc25a4-specific siRNAs were designed and synthesized by GenePharma (Shanghai, China). To knock down the target genes, siRNAs at a final concentration of 50 nM were transfected into N2a cells according to the manufacturer’s instructions.

Slc25a4-specific siRNAs: No. 1: 5′-GUGCAGAGAAGCAGUACAATT-3′; No. 2: 5′-CCUUCAAAGACAAGUACAATT-3′; No. 3: 5′-GAUCGCCAUAAGCAGUUCUTT-3′.

### Quantitative real-Time PCR

N2a cells were transfected with siRNAs for Slc25a4 and collected for RNA isolation. RNA quality was assessed by using a NanoDrop 2000 (Thermo Scientific). The cDNAs were synthesized by ReverTra Ace qPCR RT MasterMix (Toyobo, FSQ-201) or First-Strand cDNA Synthesis Kit (Toyobo, FSK-101). qPCR was performed using SYBR Green Supermix (Bio-Rad, 172–5124).

The primer sets used in this study are listed here: β-actin-F: AGGTGACAGCATTGCTTCTG; β-actin-R: GCTGCCTCAACACCTCAAC; Slc25a4-F: TGATTGTGTCGTGAGAATCCCC; Slc25a4-R: AGAACTGCTTATGGCGATCCA.

### Transmission electron microscopy

N2a cells were transfected with the vector plasmids or CVS-M plasmids, and then, cells were fixed with 2.5% glutaraldehyde in 0.1 M sodium phosphate buffer (pH 7.4) for 4 h at room temperature. The cells were then fixed in 1% osmium tetroxide, dehydrated stepwise with graded ethanol, and embedded in epoxy resin (Sigma). Next, 60-nm- to 80-nm-sized ultrathin sections were obtained and then stained with uranyl acetate and lead citrate. Images were taken with an H-7650 transmission electron microscope (Hitachi Ltd., Japan).

### Fluorescence recovery after photobleaching

N2a cells were seeded on circular slides and placed in a 12-well plate and transfected with pDsRed2-ER plasmids to stain ER; cells were infected with CVS-P-GFP at an MOI of 1 or mock-infected at 12 h post-transfection. At 24 h post-infection, the photobleaching was performed using the Vector photomanipulation module attached to a Marianas spinning disk confocal platform (3i, Denver, Colorado). The images were acquired using a Plan-Apochromat 100×/1.4 oil lens (Carl Zeiss, Jena, Germany). The incubation system (Okolab, Naples, Italy) was set at 5% CO_2_ and 37°C. Images were acquired with the 488-nm and 594-nm laser.

For data analysis, mean background fluorescence was measured from an area outside the cells and subtracted from other measurements. Mean fluorescence intensities of each photobleached area were also corrected for the photobleaching that occurred during image acquisition post-bleach and normalized by the average fluorescence pre-bleach. Photobleaching post-bleach was measured on non-bleached compartments. After normalization, mean recovery was calculated using EasyFRAP (49) and fit with a double-exponential model: *Y*(*t*) = *Y*0 + *A*fast(1 − *e* − *K*fast * *t*) + *A*slow(1 − *e* − *K*slow * *t*).

### Construction of the recombinant RABV

The recombinant plasmid pcDNA3.1-CVS was constructed by inserting the genome of CVS-B2c into the mammalian expression vector pcDNA3.1 as described previously (50). DNA fragments including CVS-M were amplified from the recombinant plasmid pcDNA3.1-CVS using the Phanta Max Super-Fidelity DNA polymerase (Vazyme Biotech, P505-d1). A glycine-to-glutamic acid point mutation was then introduced on amino acid 57 of CVS-M. The primer sets used were designed by Primer 6 (PREMIER Biosoft Biolabs) (Table 2). The recombinant plasmid pcDNA3.1-CVS was digested with BlpI/PacI, and the cloning was performed using the ClonExpress II One Step Cloning Kit (Vazyme Biotech, C112-01). The sequences of all constructs were verified by sequencing.

**TABLE 2:** Primers used for recombinant RABV construction^*a*^

Primers	Sequences (5′−3′)
pcDNA3.1-CVS-M-F	agtcgaggctgacaagctaagcAAAATCATGCAAGATGATTTGAATCG
pcDNA3.1-CVS-M-R	tgaggaaccatctgtttaattaaGTCTTTTGAGGGATGTTAATAGTTTTTT
G57E-F	CTTTTGTGTCAACGAGGAGGTTAAAGCGTG
G57E-R	CACGCTTTAACCTCCTCGTTGACACAAAAG

^*a*^ Lowercase letters represent recombinant homology arm sequences.

### Rescue of the mutant RABV

Recombinant RABVs were rescued as reported previously (46). Briefly, N2a cells were transfected with 2 µg of a fully infectious clone (pcDNA3.1-CVS or pcDNA3.1-CVS-M-G57E), 0.5 µg of pcDNA3.1-N, 0.25 µg of pcDNA3.1-P, 0.15 µg of pcDNA3.1-G, and 0.1 µg of pcDNA3.1-L using jetPRIME (Polyplus) according to the manufacturer’s instruction. Six days post-transfection, supernatants were harvested and examined for the presence of rescued viruses using FITC-conjugated anti-RABV P antibody (prepared in our laboratory). The rescued virus was propagated and titrated in N2a cells.

### Scanning electron microscopy

N2a cells were seeded on circular slides and placed in a 12-well plate and then infected with different RABV strains or its mutant for 36 h, and then, cells were fixed with 2.5% glutaraldehyde in 0.1 M sodium phosphate buffer (pH 7.4) for 4 h at room temperature. The slides were washed with ultrapure water and were sputtered with gold. Images were taken with an SU8010 scanning electron microscope (Hitachi Ltd., Japan). SEM analysis with 4,000× and 15,000× magnification was performed to study the cellular morphology.

### Mouse infection

Female Thy1-GFP (6-week-old, *n* = 9) mice were i.c. inoculated with 25 µL of CVS (20 FFU), DRV (200 FFU), or CVS-M-G57E (200 FFU). At 6 d.p.i., mice were euthanized with CO_2_. After perfusion with 4% paraformaldehyde, the brains were removed and postfixed in freshly prepared 4% paraformaldehyde for 24 h. The brains were cut into 50-µm serial sections on a vibratome for observation. Fluorescent images were captured under an EVOS FL Auto imaging system (Thermo Fisher Scientific, USA).

### Statistical analysis

The quantities and ratios of differentially expressed proteins and comparisons of biological tests were analyzed and represented by using GraphPad Prism (version 8.0) software (GraphPad, San Diego, CA, USA). The western blot graphs were analyzed using ImageJ software. The data were expressed as the mean and standard deviation. The asterisks indicate statistical significance (*P < 0.05, **P < 0.01, and ***P < 0.001).

## SUPPLEMENTAL MATERIAL

Supplemental figures mbio.02880-23-s0001.docx Fig. S1 to S4.

ASM does not own the copyrights to Supplemental Material that may be linked to, or accessed through, an article. The authors have granted ASM a non-exclusive, world-wide license to publish the Supplemental Material files. Please contact the corresponding author directly for reuse.
