# Supplementary material for: Lyssavirus M protein degrades neuronal microtubules by reprogramming mitochondrial metabolism
Source: mBio. 2024 Feb 13;15(3):e02880-23. doi: 10.1128/mbio.02880-23 (PMC10936203; doi:10.1128/mbio.02880-23)
Supplement: Supplemental figures — Fig. S1 to S4. [file mbio.02880-23-s0001.docx]

**Supplementary Figures for**

**Lyssavirus M protein degrades neuronal microtubules by reprogramming mitochondrial metabolism**

Yueming Yuan^1,4#^, An Fang^1,4#^, Haoran Wang^1,4^, Caiqian Wang^1,4^, Baokun Sui^1,4^, Jianqing Zhao^1,4^, Zhen F. Fu^1,4^, Ming Zhou^*1,4^, Ling Zhao^*1,2,3,4^

^1^ State Key Laboratory of Agricultural Microbiology, Huazhong Agricultural University, Wuhan 430070, China

^2^ Hubei Hongshan Laboratory, Wuhan 430070, China

^3^ Frontiers Science Center for Animal Breeding and Sustainable Production, Wuhan 430070, China

^4^ Key Laboratory of Preventive Veterinary Medicine of Hubei Province, College of Veterinary Medicine, Huazhong Agricultural University, Wuhan 430070, China

# Yueming Yuan and An Fang contributed equally to this work.

*Corresponding author: Ming Zhou, State Key Laboratory of Agricultural Microbiology, Huazhong Agricultural University, Wuhan, 430070, China. E-mail: [mingzhou@mail.hzau.edu.cn](mailto:mingzhou@mail.hzau.edu.cn); Ling Zhao, State Key Laboratory of Agricultural Microbiology, Huazhong Agricultural University, Wuhan, 430070, China. E-mail: [zling604@outlook.com](mailto:zling604@outlook.com), [lingzhao@mail.hzau.edu.cn](mailto:lingzhao@mail.hzau.edu.cn)


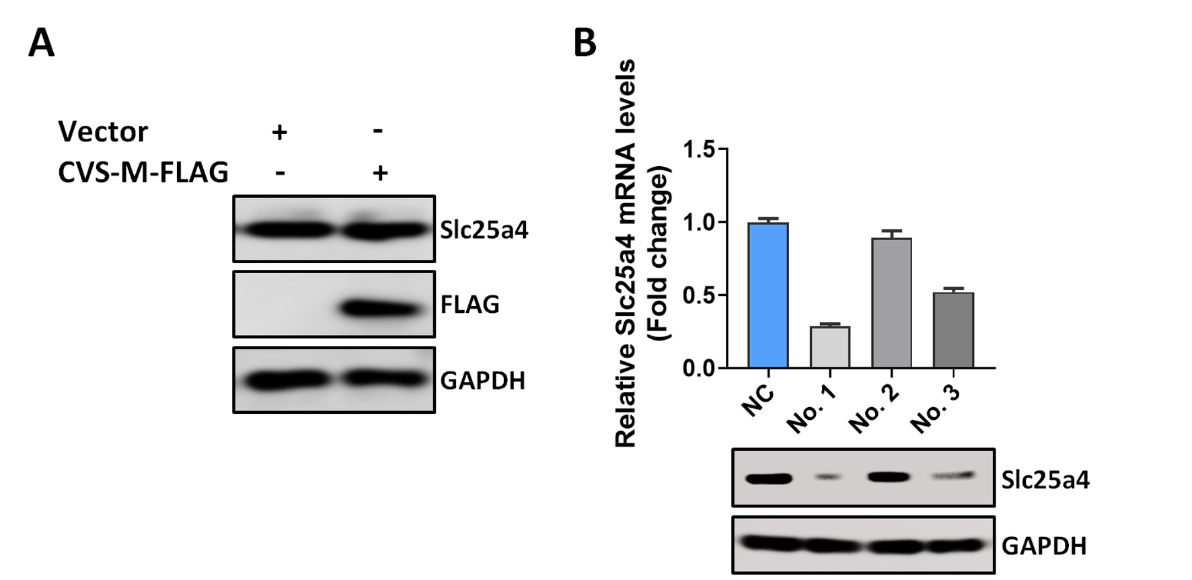


**Figure S1. CVS-M does not affect Slc25a4 expression. Slc25a4 knockdown with siRNAs, related to Figure 1.**

**(A)** N2a cells were transfected with Vector or CVS-M-FLAG for 36 h. Cell lysates were analyzed by WB.

**(B)** Three pairs of siRNA primers for Slc25a4 were transfected into N2a cells for 36 h. Then the total RNA in cells was isolated and analyzed for Slc25a4 mRNA level by Real-Time Quantitative Reverse Transcription PCR (qPCR), or the cell lysates were analyzed by WB with Abs for Slc25a4 and GAPDH. Error bars, mean ± SD of three experiments.


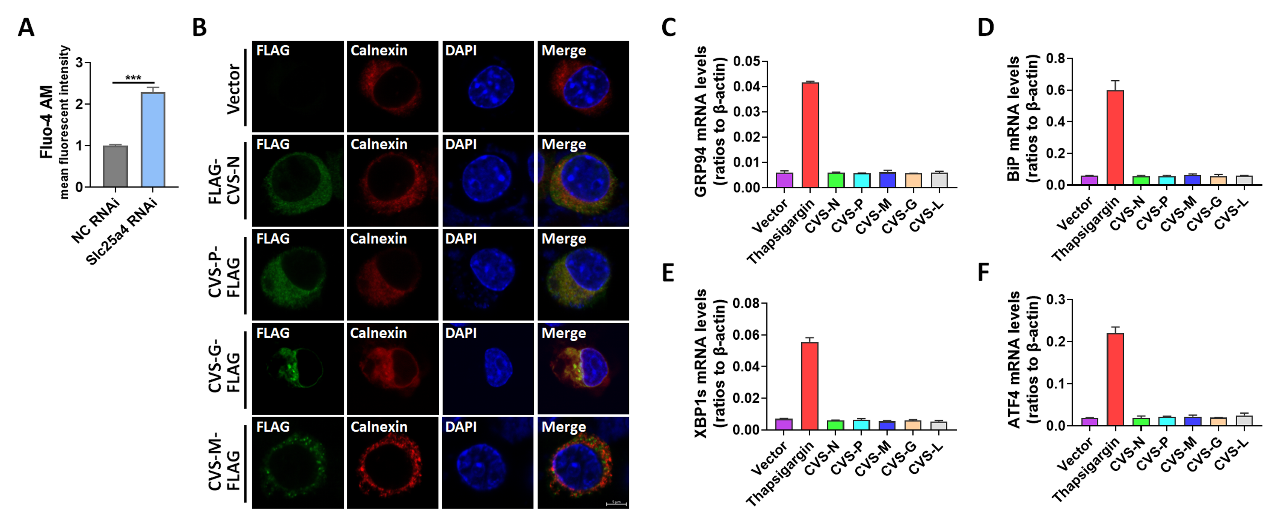


**Figure S2. Knockdown of Slc25a4 causes an increase in cellular Ca^2+^ concentration. Expression of CVS structural proteins does not induce ER stress. CVS-M-FLAG causes a change in ER morphology in N2a cells. Related to Figure 2.**

**(A)** N2a cells were transfected with siRNAs for Slc25a4 for 36 h, then stained with Fluo-4 AM and Ca^2+^ concentrations were measured.

**(B)** N2a cells were transfected with Vector, FLAG-CVS-N, CVS-P-FLAG, CVS-G-FLAG, or CVS-M-FLAG for 36 h, then fixed and stained with Calnexin to observe ER morphology. Scale bar, 5μm.

**(C-F)** N2a cells were transfected with Vector, CVS-N, CVS-P, CVS-M, CVS-G, and CVS-L for 36 h, or treated with Thapsigargin (an ER stress activator capable of inducing unfolded protein response (UPR), introduced as a positive control, 50 nM working solubility) for 24 h, qPCR was used to measure GRP94 (C), BiP (D), XBP1s (E), and ATF4 (F) mRNA levels.

Error bars, mean ± SD of three experiments. Statistical analysis of comparisons between groups was carried out by Student’s t-test (*, P<0.05; **, P<0.01; ***, P<0.001; ns, not significant).


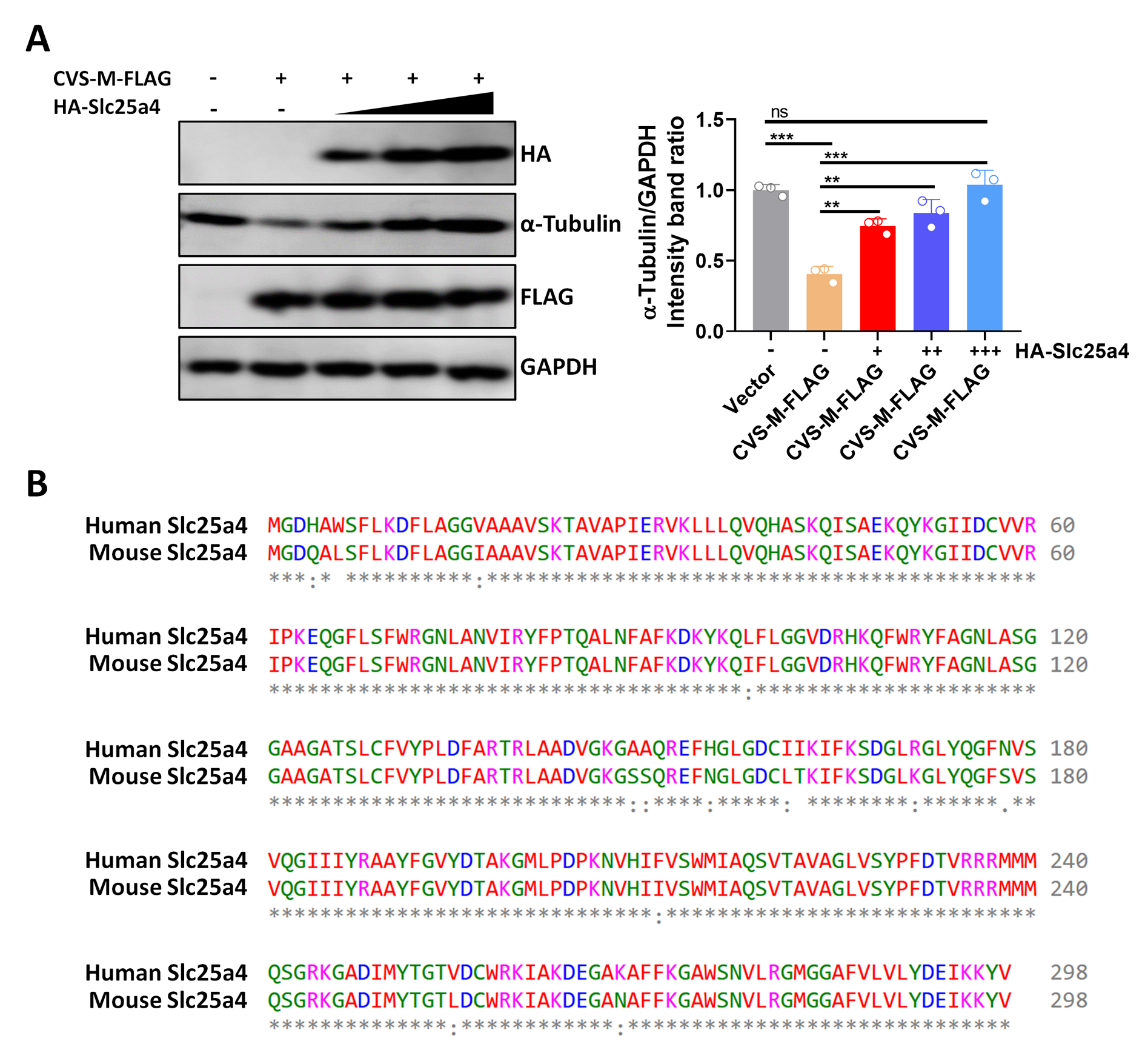


**Figure S3. Exogenous Slc25a4 can rescue the degradation of α-tubulin caused by CVS-M overexpression. Sequence comparison between human Slc25a4 and mouse SLc25a4. Related to Figure 3.**

**(A)** CVS-M-FLAG and the indicated volumes of HA-Slc25a4 were coexpressed in N2a cells, and lysates were analyzed by WB.

**(B)** The amino acid sequences of human Slc25a4 and mouse Slc25a4 were compared using Clustal Omega (https://www.ebi.ac.uk/Tools/msa/clustalo/)

Error bars, mean ± SD of three experiments. Statistical analysis of comparisons between groups was carried out by Student’s t-test (*, P<0.05; **, P<0.01; ***, P<0.001; ns, not significant).


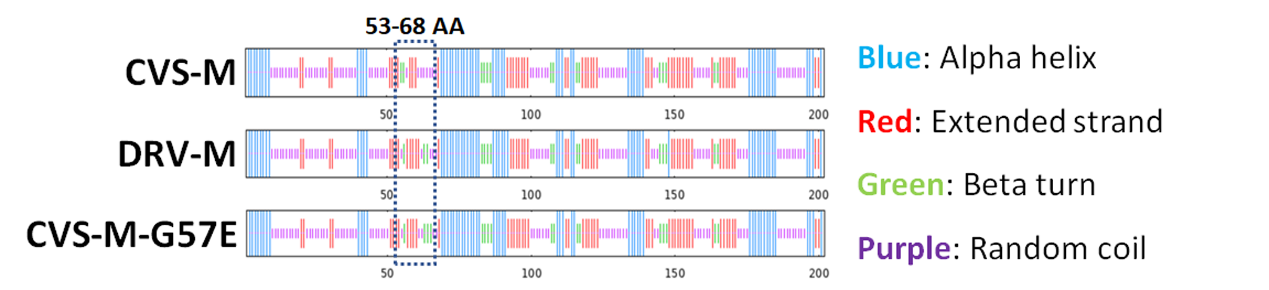


**Figure S4. Comparison of the protein secondary structures of CVS-M, DRV-M and CVS-M-G57E.**

The improved self-optimized prediction method (SOPMA) online tool (https://npsa-pbil.ibcp.fr/cgi-bin/npsa_automat.pl?page=npsa_sopma.html) was used to predict and compare the protein secondary structures of CVS-M, DRV-M and CVS-M-G57E.
